# Gross tumour volume radiomics for prognostication of recurrence & death following radical radiotherapy for NSCLC

**DOI:** 10.1038/s41698-022-00322-3

**Published:** 2022-10-27

**Authors:** Sumeet Hindocha, Thomas G. Charlton, Kristofer Linton-Reid, Benjamin Hunter, Charleen Chan, Merina Ahmed, Emily J. Greenlay, Matthew Orton, Catey Bunce, Jason Lunn, Simon J. Doran, Shahreen Ahmad, Fiona McDonald, Imogen Locke, Danielle Power, Matthew Blackledge, Richard W. Lee, Eric O. Aboagye

**Affiliations:** 1 Lung Unit, The Royal Marsden NHS Foundation Trust, London, UK; 2 AI for Healthcare Centre for Doctoral Training, Imperial College London, London, UK; 3 Institute of Cancer Research NIHR Biomedical Research Centre, London, UK; 4 Cancer Imaging Centre, Department of Surgery & Cancer, Imperial College London, London, UK; 5 Guy’s Cancer Centre, Guy’s and St Thomas’ NHS Foundation Trust, London, UK; 6 Lung Unit, The Royal Marsden NHS Foundation Trust, Sutton, UK; 7 Clinical Trials Unit, Royal Marsden NHS Foundation Trust, Sutton, UK; 8 Artificial Intelligence Imaging Hub, Royal Marsden NHS Foundation Trust, Sutton, UK; 9 Department of Clinical Oncology, Charing Cross Hospital, London, UK; 10 Radiotherapy and Imaging, Institute of Cancer Research, London, UK; 11 Early Diagnosis and Detection Team, Institute of Cancer Research, London, UK

**Keywords:** Non-small-cell lung cancer, Cancer imaging, Cancer models, Radiotherapy, Tumour biomarkers

## Abstract

Recurrence occurs in up to 36% of patients treated with curative-intent radiotherapy for NSCLC. Identifying patients at higher risk of recurrence for more intensive surveillance may facilitate the earlier introduction of the next line of treatment. We aimed to use radiotherapy planning CT scans to develop radiomic classification models that predict overall survival (OS), recurrence-free survival (RFS) and recurrence two years post-treatment for risk-stratification. A retrospective multi-centre study of >900 patients receiving curative-intent radiotherapy for stage I-III NSCLC was undertaken. Models using radiomic and/or clinical features were developed, compared with 10-fold cross-validation and an external test set, and benchmarked against TNM-stage. Respective validation and test set AUCs (with 95% confidence intervals) for the radiomic-only models were: (1) OS: 0.712 (0.592–0.832) and 0.685 (0.585–0.784), (2) RFS: 0.825 (0.733–0.916) and 0.750 (0.665–0.835), (3) Recurrence: 0.678 (0.554–0.801) and 0.673 (0.577–0.77). For the combined models: (1) OS: 0.702 (0.583–0.822) and 0.683 (0.586–0.78), (2) RFS: 0.805 (0.707–0.903) and 0·755 (0.672–0.838), (3) Recurrence: 0·637 (0.51–0.·765) and 0·738 (0.649–0.826). Kaplan-Meier analyses demonstrate OS and RFS difference of >300 and >400 days respectively between low and high-risk groups. We have developed validated and externally tested radiomic-based prediction models. Such models could be integrated into the routine radiotherapy workflow, thus informing a personalised surveillance strategy at the point of treatment. Our work lays the foundations for future prospective clinical trials for quantitative personalised risk-stratification for surveillance following curative-intent radiotherapy for NSCLC.

## Introduction

Lung cancer is the leading cause of cancer mortality globally^1^, of which 85% is non-small cell lung cancer (NSCLC)^2^. Radiotherapy is a key treatment modality for NSCLC, used when surgery is not appropriate due to the tumour location or patient’s fitness. Recurrence occurs in up to 36% of NSCLC patients treated with curative-intent radiotherapy^3^. Detecting recurrence early may facilitate further (curative) treatment and improve overall survival (OS). Post-treatment surveillance is variable in frequency and imaging-modality used. High-quality evidence to inform optimal surveillance strategies post-treatment is lacking and, the UK’s National Institute for Healthcare and Clinical Excellence have called for further research to develop risk-stratification models to determine the optimal follow-up pattern^4^.

TNM-stage is currently the gold-standard prognosticator^5,6^ however outcomes within each TNM-stage group vary considerably, highlighting the need for more accurate tools^1^. Radiomics, the use of multitudinous data-characterisation algorithms to comprehensively quantify tumour phenotype, has demonstrated prognostic value in numerous studies^7–10^. As a non-invasive biomarker it may herald a paradigm-shift in personalised medicine.

Several studies have employed CT or PET-CT radiomics to predict OS in large heterogeneous post-radiotherapy NSCLC cohorts with good results (AUC 0.65–0.66)^2,8,11–14^. Arshad et al. identified a PET-radiomics feature vector that was able to predict a 14-month survival difference. The feature vector was independent of known prognostic factors, such as stage and tumour volume, and invariant to the type of PET/CT manufacturer^1^. While OS is a less ambiguous and often more readily obtained endpoint, we believe recurrence and recurrence-free survival (RFS) are more clinically useful as predicting the timepoint of recurrence may facilitate the earliest possible introduction of the next line of treatment. Studies with smaller cohorts looking specifically at Stage III patients or those treated with SBRT have been undertaken with mixed results^15–18^.

Feature reduction is often required prior to radiomics modelling owing to the large number of available features contrasted with relatively small clinical datasets. The best combination of feature reduction and machine learning classifier is often data and task-dependent, thus warranting a comparison of several viable feature reduction and classifier combinations.

We hypothesised that radiomic features extracted from radiotherapy planning CT gross tumour volumes (GTVs) could be used to predict OS, RFS, and recurrence at two years from treatment and risk-stratify patients. GTV definition is based on international guidelines and subject to peer-review, thus representing a robust and readily available structured dataset^19,20^. To the best of our knowledge only one study has used GTV as the volume of interest for radiomics, however specific utility of GTV-derived radiomics was not discussed and the study only looked at OS^8^. Such models could be integrated into the routine radiotherapy workflow, thus informing a personalised surveillance strategy at the point of treatment. We compared 11 machine learning algorithms and 9 feature reduction techniques to determine the best combination for prediction of each endpoint.

## Results

### Characteristics of data and patients

**Fig. 1:**
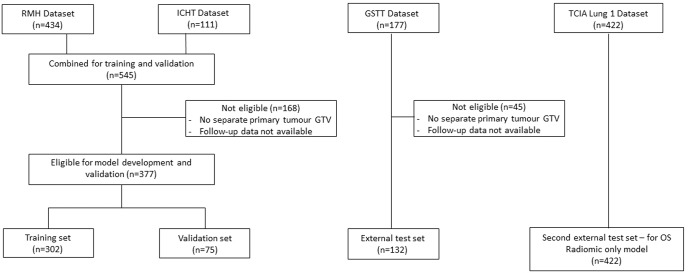
Datasets used for the study. The RMH and ICHT Datasets were combined and eligible patients (*n* = 377) were divided with an 80:20 ratio for training and validation. Eligible patients from the GSTT dataset (*n* = 132) were used for external testing. The TCIA Lung 1 dataset (*n* = 422) was used as a second external test set for the radiomic-OS model. Ineligible patients were those lacking full follow-up data or where the GTV did not encompass only the primary tumour.

A total of 931 patients were eligible for inclusion. A total of 509 eligible patients from the Royal Marsden (RMH), Imperial College Healthcare (ICHT) and Guy’s and St Thomas’ (GSTT) Datasets were split into training (*n* = 302), validation (*n* = 75) and external test sets (*n* = 132) and a further 422 patients from the TCIA Lung 1 dataset were used as a second external test set for the radiomic-OS model, as described above (Fig. 1). Median follow-up was 762 days. Patient demographics and clinical parameters are summarised in Table 1.

**Table 1:** Demographic and clinical parameters for combined training-validation and external test sets.

Parameter	Combined Training & Validation Sets n = 377	External Test Set *n* = 132	*P*-value
Age (IQR) years	76 (12)	73 (13)	<0.001
Sex (% of *n*)			0.854
Male	205 (54.4)	55.3	
Female	172 (45.6)	59 (44.7)	
WHO Performance Status (% of *n*)			0.215
0	58 (15.4)	11.3	
1	210 (55.7)	52.3	
2	109 (28.9)	48 (36.4)	
Body Mass Index (IQR)	25.5 (6.2)	26.45 (7.5)	0.093
Smoking Status (% of *n*)			0.552
Never	35 (9.3)	7.6	
Ever	342 (90.7)	122 (92.4)	
TNM8 T stage (% of *n*)			0.506
1	181 (48.0)	55.3	
2	110 (29.2)	26.5	
3	46 (12.2)	9.1	
4	40 (10.6)	12 (9.1)	
TNM8 N stage (% of *n*)			0.109
0	277 (73.5)	9.5	
1	17 (4.5)	(0.8)	
2	67 (17.8)	13.6	
3	16 (4.2)	8 (6.1)	
TNM8 Overall stage (% of *n*)			0.072
1	197 (52.3)	63.6	
2	62 (16.4)	11.4	
3	118 (31.3)	33 (25)	
FEV1, percent predicted (IQR)	73.2 (34)	69 (35.25)	0.04
TLCO, percent predicted (IQR)	58 (23)	56 (29)	0.206
Days from planning scan to first fraction (IQR)	18 (7)	18(6)	0.789
Size of primary (IQR)	29 (21)	27 (20.5)	0.253
SUV primary (IQR)	9.1(8.5)	8.65 (7.65)	0.999
Max nodal SUV (IQR)	6.7 (6.25)	6.4 (3.8)	0.084
Nodal avidity (% of n)			0.296
Yes	102 (27.1)	42(31.8)	
No	275 (72.9)	90 (68.2)	
Nodal Sampling (% of n)			0.269
Yes	101 (26.8)	31.8	
No	276 (73.2)	90 (68.2)	
Histology (% of n)			0.012
Adenocarcinoma	170 (45.1)	38.6	
Squamous	112(29.7)	31.1	
Other	29 (7.7)	3(2.3)	
No pathology	66 (17.5)	37 (28)	
Treatment type (% of n)			0.174
SBRT	174 (46.1)	54.5	
Conventional RT	98 (26)	18.9	
Chemo + RT	105 (27.9)	35 (26.5)	
Number of fractions (IQR)	20 (15)	8 (19.75)	0.962
Total Dose, Gy (IQR)	55 (5)	55 (5.4)	0.017
Biologically Effective Dose, Gy (IQR)	79.2 (45.4)	105 (38.7)	0.219
Planning Target Volume, cm^3^ (IQR)	103.65 (246.3)	91·4 (238.7)	0.524
Recurrence at 2 years (% of n)	137 (36.3)	40 (30.3)	0.21
Recurrence or death at 2 years (% of n)	181 (48)	58 (43.9)	0.42
Death at 2 years (% of *n*)	127 (33.7)	43 (32.6)	0.816
Median length of follow-up (range) days	739 (33–2358)	785 (26–1442)	

Features not used for modelling are not shown. Categorical data are summarised with means and percentages and *p*-values pertain to Fishers exact test. Continuous data are summarised with median and inter-quartile range (IQR) and *p*-values pertain to Wilcoxon rank sum test.

Training-validation and external test set median age was 76 and 73 respectively, and recurrence, RFS and OS rates at two years were 36·3% vs 30·3%, 48% vs 43·9% and 33.7% vs 32.6%, respectively. The external test set had a higher proportion of patients with earlier stage disease (TNM8 T1-stage 55.3% vs 48%, N0-stage 79.5% vs 73.5%), treated with SBRT (54.5% vs 46.1%), with a radiological diagnosis (28% vs 17.5%) and less patients with adenocarcinoma (38.6% vs 45.1%)

**Fig. 2:**
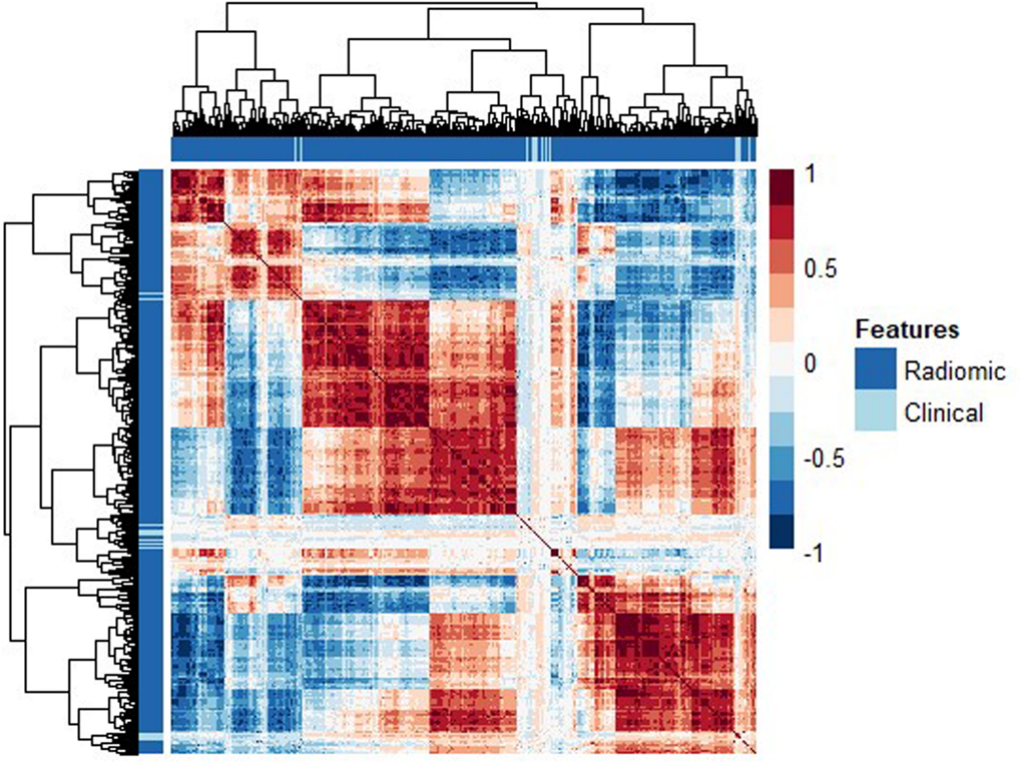
A hierarchical clustering heatmap based on training set data shows correlation between radiomic and clinical features, with all features on the X and Y axes respectively. Axis trees show a number of groups based on sample-wise similarities.

A hierarchical clustering heatmap (Fig. 2) generated from the training data indicated multi-collinearity suggesting feature reduction could be undertaken without resultant loss of useful information.

**Fig. 3:**
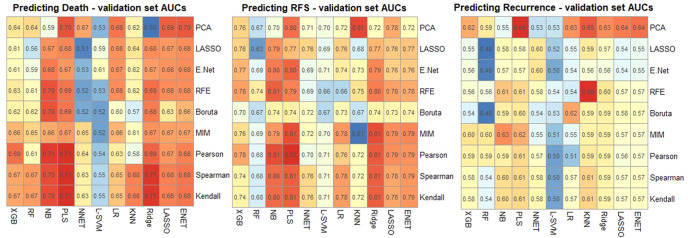
Heatmaps illustrating the performance of each machine learning algorithm (columns) with each feature reduction technique (rows), measured by validation set AUC. PCA Principle Component Analysis, LASSO Least Absolute Shrinkage and Selection Operator, E Net Elastic-Net, RFE Recursive Feature Elimination, MIM Mutual Information, XGB Extreme Gradient Boosting machine, RF Random Forest, NB Naïve-Bayes, PLS Partial Least Squares, NNET Neural Network, L-SVM Linear Support Vector Machine, LR Logistic regression, KNN K-Nearest Neighbours, Ridge Ridge regression. The best performing models were, for OS Spearman correlation with PLS, for RFS Pearson correlation with PLS and for recurrence: PCA with an ensemble of PLS, KNN and E Net.

Heatmaps in Fig. 3 showing validation set AUCs illustrate the results of our experiments combining feature reduction techniques and machine learning algorithms for each endpoint. When averaging across the machine learning algorithms (columns), the feature reduction techniques (rows) resulting in the highest AUCs were: Spearman correlation, Pearson correlation, and Principle Component Analysis for OS, RFS and recurrence respectively (Fig. 3). The final prediction models chosen were: For OS and RFS, PLS alone; for recurrence, an ensemble of PLS, KNN, and Elastic-Net regression.

The list of radiomic features surviving feature reduction for OS and RFS, and the top 15 features contributing to principal components for recurrence are indicated in Supplementary Tables 5 and 6.

**Fig. 4:**
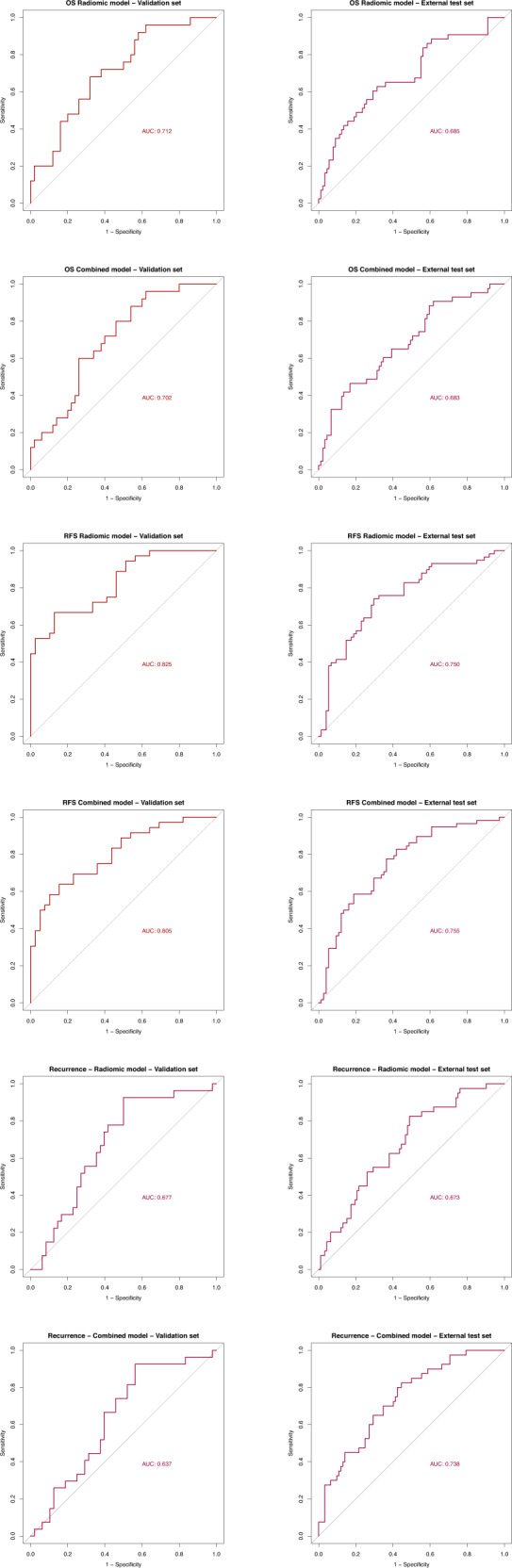
ROC curves for the validation and external test sets for the Radiomic and Combined models for each prediction. The final models were, for OS: Spearman correlation with PLS, for RFS: Pearson correlation with PLS and for recurrence: PCA with an ensemble of PLS, KNN and Elastic Net.

Table 2 and Fig. 4 illustrate validation and external test set results for our models compared to the TNM model. For OS, the Radiomic model consistently demonstrated superior AUC across the validation and external test set and also performed well on the second external Lung 1 dataset.

**Table 2:** AUC with 95% confidence intervals for the validation and external test set(s) for each prediction model, benchmarked against a model based on TNM-stage.

Out-come	Model	Validation set results			External test set result			NSCLC-radiomics lung 1 dataset result	
		AUC	95% CI	*p*-value	AUC	95% CI	*p*-value	AUC	95% CI
OS	Radiomic	0.712	0.592–0.832	0.013	0.685	0.585–0.784	0.621	0.64	0.587–0.694
	Combined	0.702	0.583–0.822	0.007	0.683	0·586–0.78	0.597		
	Clinical	0.627	0.494–0.76	0.204	0.674	0.579–0.77	0.725		
	TNM	0.573	0.442–0.704		0.663	0.57–0.759			
RFS	Radiomic	0.825	0.733–0.916	0.015	0.750	0.665–0.835	0.103		
	Combined	0.805	0.707–0.903	0.008	0.755	0.672–0.838	0.037		
	Clinical	0.705	0.582–0.829	0.880	0.682	0.587–0.777	0.869		
	TNM	0.711	0.597–0.824		0.677	0.59–0.765			
Rec	Radiomic	0.678	0·554–0.801	0.053	0.673	0.577–0.77	0.833		
	Combined	0.637	0.51–0.775	0.084	0.738	0.649–0.826	0.131		
	Clinical	0.601	0·47–0.732	0.238	0.678	0.582–0.774	0.391		
	TNM	0.551	0·42–0.682		0.683	0.596–0.77			

*P*-values compare each model to the TNM-model.

Despite overlap in confidence intervals, the Radiomic and Combined models were significantly superior to the TNM model in the validation set at the 5% significance level (*p*-values 0.013 and 0.007, respectively). This was not seen in the external test set, however. For RFS, the Combined model was significantly superior to TNM in the external test set (AUC 0.755 vs 0.677, *p*-value 0.037).

For recurrence, the Radiomic and Combined models were significantly superior to TNM in the validation set, however this did not extend to the external test set.

**Fig. 5:**
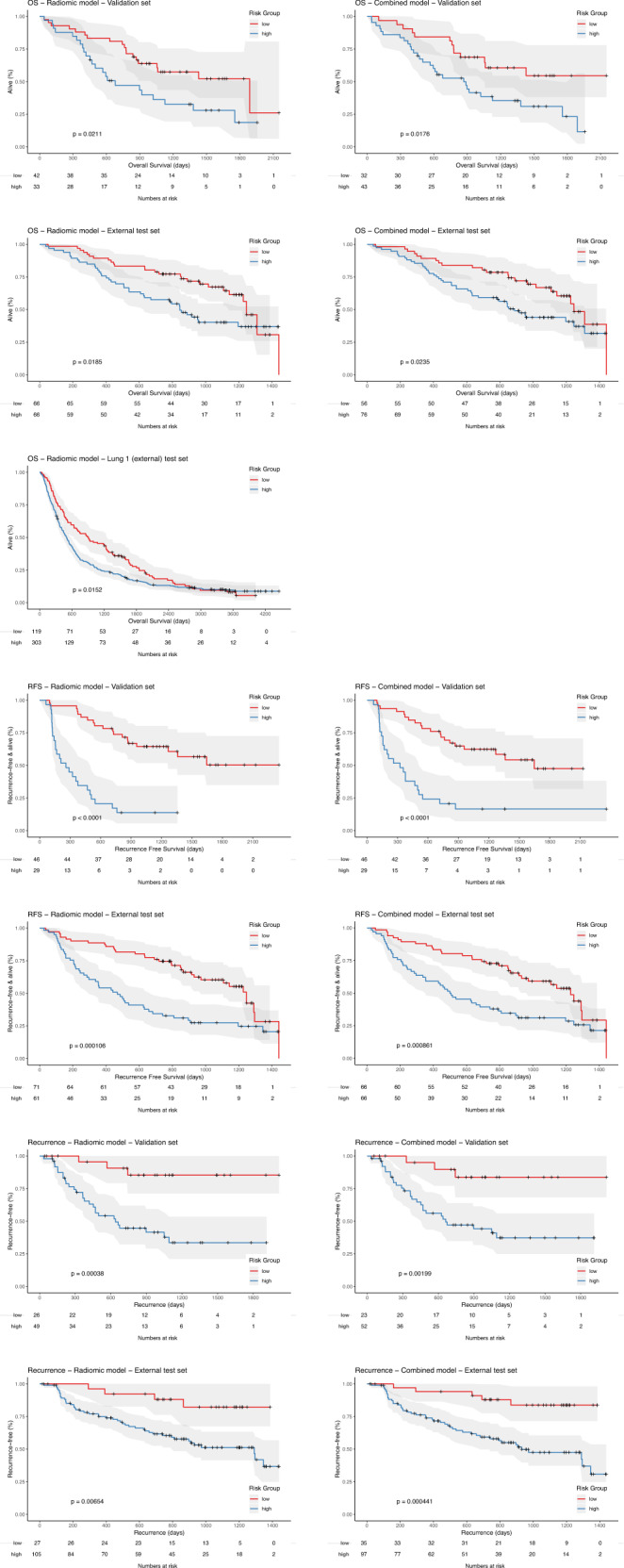
Kaplan Meier survival curves for low and high-risk groups in both validation and external test set(s) for the Radiomic and Combined models for OS, RFS and recurrence. *P*-values correspond to log-rank tests. Curves demonstrate good separation between high and low risk groups with log-rank tests confirming a statistically significant difference for prediction of each endpoint at the 5% level.

Validation and external test set Kaplan Meier curves for each endpoint are shown in Fig. 5 and demonstrate good separation between high and low-risk groups with log-rank tests confirming a statistically significant difference for prediction of each endpoint at the 5% level. For OS-risk-stratification, Kaplan-Meier curves based on the external test set Radiomic and Combined models demonstrate a median survival difference of >300 days. In the Lung 1 dataset for the Radiomic model this extends to >400 days. For RFS, Radiomic and Combined models demonstrate a median difference in the external test set of >700 days, and for recurrence, Kaplan Meier curves again demonstrate good risk-stratification ability with <25% of the low-risk group experiencing recurrence over the study period. Using the same threshold as for Kaplan-Meier analysis, classification was performed for each endpoint (Supplementary Table 7). Classification metrics including Balanced Accuracy, F1 score, sensitivity, specificity, positive and negative predictive value along with Brier scores and calibration curves are detailed in Supplementary Tables 7, 8 and Supplementary Fig. 3.

### Lymph node models

Validation set AUCs are presented in Table 3 and show that for predicting OS, a model built on lymph node radiomic features does not outperform or enhance the primary tumour model, however for recurrence and RFS, integration of nodal features is seen to improve model performance. Similar results have been demonstrated for predicting OS with PET-CT derived nodal features in NSCLC, and loco-regional control with CT-derived nodal features in head and neck cancer^21,22^, however we believe we are the first to report on OS, RFS and recurrence in NSCLC using CT-derived nodal radiomics. This dataset is small, and more data is required before we can draw meaningful conclusions, but this does suggest that there is merit in developing imaging models that utilise more than just the primary tumour in outcome prediction.

**Table 3:** Validation set AUC and 95% CIs for models built using radiomic features extracted from the primary tumour only, largest nodal volume only, and these features combined.

Endpoint	Metric	Tumour	Nodal	Tumour + Nodal
OS	AUC	0.72	0.453	0.573
	95% CI	0.513–0.927	0.203–0.703	0.329–0.82
RFS	AUC	0.679	0.582	0.746
	95% CI	0.464–0.893	0.347–0.82	0.55–0.941
Rec	AUC	0.583	0.571	0.622
	95% CI	0.347–0.82	0.329–0.812	0.394–0.85

Models were produced for each clinical endpoint.

## Discussion

In this multicentre study of more than 900 patients treated with curative-intent radiotherapy for NSCLC, we have explored multiple machine learning classifiers and feature reduction techniques using radiomics to develop, validate and externally test prognostic models for three different clinical endpoints—OS, RFS and recurrence at two years from the start of treatment. Previous studies for imaging-based NSCLC prognostication have focused on univariate or multivariate survival analysis using Cox Proportional Hazards models with a small number of features^23^. Cox models require linearity of each variable and that the proportional hazards assumption is met. This can be challenging in the context of real-world data, leading to inappropriate model fit^24^. Here we adopt a predictive modelling approach using a large radiomic and clinical feature set for prognostication. The two year endpoint is relevant for NSCLC and has been previously used for prognostication studies^11,25^.

Typically, radiomic models require manual segmentation, however by utilising the GTV contoured for radiotherapy planning as the VOI, our models avoid this added work. As GTV segmentation is supported by international guidelines and best-practice guidance advocates peer-review, it provides a readily available structured dataset for feature extraction^19,20^. Such models could be integrated into the routine radiotherapy workflow and may demonstrate future utility in personalised surveillance stratification, whereby those at higher risk have the more intensive follow-up. Those identified as lower risk could have lower intensity follow-up and frequency of CT imaging resulting in less radiation exposure, time burden and potentially anxiety for patients, and less healthcare resource demand. A similar risk-stratified surveillance programme has been described for post-surgical management of NSCLC^26,27^.

An additional reason for using radiotherapy GTVs is that they are determined using more than just macroscopic CT appearances. In addition, clinician experts consider patient characteristics, patterns of tumour spread pertaining to the specific stage and histological tumour type, as well as information from other sources e.g., EBUS and PET results. Though utilisation of semi and fully automated contouring software may provide a more objective VOI delineation, they are not able to capture this nuanced clinical information and are therefore unlikely to imminently replace clinician segmentations. While AI models to auto-derive GTV are in development, these are commonly trained on clinician GTVs as ground-truth and are likely to still require clinical augmentation or sign-off prior to use^28^. A model that does not use GTVs as input would therefore not be readily deployable in the healthcare setting at the point of radiotherapy planning, which is the goal of our model.

AUCs for the Radiomic and Combined models were consistent between validation and external test sets for OS and RFS. For recurrence, the combined model may demonstrate underfitting in the validation set. Interestingly, the combination of clinical data does not improve model performance markedly, suggesting that radiomics alone may be capturing the clinical/biological picture of disease effectively. Despite overlapping confidence intervals, performance of the Radiomic and Combined models is reasonable, demonstrating superior AUC values compared to the TNM-model. This is statistically significant in the validation set for OS and RFS and approaches significant for recurrence. Significance is however only demonstrated in the external test set for the Combined-RFS model.

The exception to TNM superiority is in the external test set for recurrence where the combined, clinical and TNM models are superior. This may be due to the external test set having a higher proportion of early-stage patients treated with SBRT, a lower number of adenocarcinoma cases and a higher number lacking pathological confirmation for NSCLC.

Deep-learning is an alternative approach to radiomics which has also shown promise in imaging-based predictive modelling. Both approaches have well-described advantages and disadvantages^10,29^. We used a radiomics method in this study due to the requirements for very large datasets associated with deep-learning. Deep-learning has been criticised for its black-box nature^10,11^. While efforts to improve explainability of deep-learning including class activation maps are in development^30^ and radiomic features themselves are not easily explainable to all clinicians and patients, radiomics does offer a quantitative, mathematical method to map from image to prediction^8^. We have observed associations between radiomic and clinical features selected for our OS, RFS and recurrence models. These include T1-stage with NGLDM coarseness for OS, and GLSZM zone percentage for recurrence (further detailed in the Supplementary Material). In addition, efforts to derive biological meaning are gaining traction, for example with genomic correlation (radiogenomics), microscopic pathological image textures and histopathologic marker expression^31^. One example of this is finding of a positive association between the radiomic feature GLCM inverse difference, included in our OS and RFS models, and hypoxia-related carbonic anhydrase (CAIX), by gene expression profiling and immunohistochemistry. Hypoxia is associated with radiation resistance and poor survival outcome in NSCLC. CAIX, a pH regulatory enzyme upregulated in hypoxia, results in an acidic tumour microenvironment^32^. This suggests our models can reflect tumour microenvironment characteristics, non-invasively.

Other strengths of our study include the multicentre design including over 900 patients from the UK and the Netherlands. Models were developed using readily available radiotherapy planning CTs and clinical data. A broad set of radiomic and clinical features were used incorporating imaging, patient demographics, fitness, tumour characteristics and treatment parameters. We also included patients treated with both SBRT and conventional (chemo)radiotherapy, thus increasing clinical utility.

Many of the selected features are multi-level separable wavelet filtered texture features. While non-separable or uni-level wavelet features may be preferable^33^, appropriate steps have been taken to remove excess features that correlate by chance, for example through removal of correlated-features, as well as inclusion of unseen validation and external test set modelling. The inclusion of validation and test datasets to mitigate unknown reproducibility concerns is also demonstrated in a recent study where Boehm et al. use wavelet derived features, specifically the HLL Coif wavelet-filtered image, which is IBSI-defined and has been found to be strongly or very strongly reproducible in multiple studies^34^. Similarly, Fotopoulou et al. validate a previously reported model comprising TexLAB-generated wavelet features, on a European patient cohort with good results^35^.

Interpolation has been shown to impact on robustness of radiomic features^36,37^. While not all features that are robust to interpolation may necessarily have clinical predictive value, given the need for feature reduction to reduce overfitting, removal of features that do not demonstrate interpolation stability may be a useful step. Detailed reporting of feature extraction methods, as described in our work, and a preliminary analysis to assess that interpolation does not affect feature reproducibility may therefore be beneficial^37,38^. While we did not undertake a preliminary analysis, we used trilinear interpolation which is considered a conservative approach^39^ and we note consistent results between our validation and external test sets.

Limitations of our work include the retrospective nature and reliance on clinical data exported from electronic healthcare records which suffers omissions. The study included patients who were treated with SBRT for presumed NSCLC without confirmed pathological diagnosis. This potentiates inclusion of patients with the benign disease or small cell lung cancer which may confound recurrence or survival rates, however our models were provided with this information, and this is reflective of real-world scenarios. Furthermore, such patients would also benefit from prognostication. We did not include patients treated with surgery pre- or post-radiotherapy, nor do we have accurate data on eligible patients that went on to receive adjuvant durvalumab or those that had oligometastatic recurrence or metachronous lung cancer that influence recurrence or survival. Future research directions include developing models built on larger homogenous datasets e.g., biopsy-proven cases treated with SBRT, or stage III only.

TexLAB 2.0, which was used for our analysis has not been compared to the IBSI digital phantom, however, our models perform well across validation and external test cohorts.

Finally, owing to the origin of the data used, while our models are reproducible, they may not be generalisable outside Europe. Testing our models in future international prospective clinical trials is warranted.

In conclusion, we have explored multiple machine learning algorithms and feature reduction techniques to develop radiomic-based prediction models for recurrence, RFS and OS two years post curative-intent radiotherapy for NSCLC. Our models can be integrated into the routine radiotherapy workflow, automating risk-stratification prior to commencement of treatment. Identifying patients at higher risk of recurrence for more intensive surveillance may facilitate earlier introduction of the next line of treatment. Our validated and externally tested models demonstrate reasonable performance with AUCs exceeding those of traditional TNM-based methods. Kaplan-Meier curves demonstrate median survival and RFS differences of >300 and >400 days respectively between low and high-risk groups. Our radiomics approach has mapped a large feature-space and may facilitate increased future explainability as new biological correlates emerge. Our work lays the foundations for future prospective clinical trials for quantitative personalised risk-stratification and surveillance following curative-intent radiotherapy for NSCLC.

## Methods

This study was reviewed by the Royal Marsden Committee for Clinical Research and approved by the UK Health Research Authority (reference number: 20/HRA/3051), ClinicalTrials.gov identifier: NCT04721444. As the data used in the study were deidentified, patient consent was not required for this type of study and as per the respective Health Research Authority and Research Ethics Council approvals.

### Datasets & pre-processing

Four datasets of patients receiving primary curative-intent radiotherapy for stage I–III NSCLC were used for this study: three independent, novel datasets (RMH, ICHT and GSTT) from UK cancer centres (the Royal Marsden NHS Foundation Trust, Imperial College Healthcare NHS Trust and Guy’s and St Thomas’ NHS Foundation Trust respectively), and the TCIA NSCLC-Radiomics Lung 1 dataset^8,40^, yielding a total of 1144 patients.

Each dataset was retrospectively collated from electronic patient record (EPR) and radiotherapy treatment planning systems (TPS) at UK National Health Service (NHS) Trusts:

- The RMH Dataset consists of 434 patients with stage I to III disease treated at The Royal Marsden NHS Foundation Trust with stereotactic or conventional radiotherapy with or without chemotherapy between 26/9/2014 and 23/10/2018.
- The ICHT Dataset consists of 111 patients with stage I to III disease treated at Imperial College Healthcare NHS Trust with conventional radiotherapy with or without chemotherapy between 3/2/2014 and 10/1/2019.
- The GSTT Dataset consists of 177 patients with stage I to III disease treated at Guy’s & St Thomas’ NHS Foundation Trust with stereotactic or conventional radiotherapy with or without chemotherapy between 21/1/2016 and 18/12/2018.

Eligible patients were those aged 18 or over and who received curative intent radiotherapy for NSCLC as determined by the local care team. Radiotherapy doses ranged from: 50–66 Gy in 3–33 fractions encompassing a range of radical stereotactic/conventional dose-fractionation schedules.

Data were collected in 2021, to allow at least 2 years of follow-up for every patient. Patients with no evidence of recurrence or death within 60 days of the 2-year endpoint, or no evidence of recurrence within 60 days of death, were labelled as having no event. 60 days was selected as the cut-off as it is an approximate time period between follow-up appointments and thus an approximate half-way point between the last time a patient was seen and the 2-year endpoint.

The following patient and tumour data were collected: age, sex, ethnicity, performance status, smoking status, body mass index, disease stage according to TNM8 and size of the primary lesion. Details on investigations and treatment were also collected, including: Standard Uptake Value (SUV) of the primary lesion and nodes based on FDG-PET-CT, details of nodal sampling, histology, lung function parameters including pre-treatment forced expiratory volume in 1 s (FEV1, as percent predicted) and diffusing capacity for carbon monoxide (TLCO, as percent predicted) and neutrophil and lymphocyte counts both prior to and after treatment, type of radiotherapy treatment administered, total dose and number of fractions, the biologically effective dose in Gy (assuming an α/β value of 10), the size of gross tumour volume (GTV) and planning target volume (PTV) and dates of the radiotherapy planning scan and first and final treatment.

Radiotherapy planning CT scans were curated and anonymised. The primary tumour GTV contoured for radiotherapy planning was used as the volume of interest (VOI) for radiomic feature extraction. As five cancer centres contributed to this study, contrast and non-contrast enhanced CT scans were acquired from scanners with different manufacturers and imaging protocols. GTVs were contoured by consultant clinical oncologists experienced in thoracic radiation oncology. Cases without a separate primary tumour GTV (i.e., where the GTV included adjacent lymph nodes) were excluded.

Images and accompanying GTV structure sets were resampled to 1 × 1 × 2.5 mm. As 2.5 mm was the median slice thickness of scans used in the study, this was felt to minimise the inference and introduction of artificial information, or information loss that is associated with upsampling or downsampling respectively^39^. Resampling to the intermediate voxel size of the dataset has also been shown to minimise interpolation artefacts and maintain robustness of radiomic features^36,37^. Trilinear interpolation was used for resampling, as this is recommended as a conservative approach in the IBSI guidelines^39^. Feature extraction was performed using our in-house texture analysis software package, TextLAB 2.0^1^. Radiomics features were extracted using 25 Hounsfield Unit intensity bins and were broadly related to volume, intensity, heterogeneity and wavelet transformations, as previously described^8^.

The patient, tumour, investigation and treatment data were also pre-processed prior to machine learning. The treatment_corr function^41^ was used to remove highly correlated features (threshold = 0.85), with Pearson correlation for continuous and Spearman correlation for categorical features. Categorical data types were the converted to numeric, using One-hot-encoding where required. Missing data were assumed to be non-dependent on outcome and missing at random. Where features had greater than a quarter of observations with missing data, these were removed. Missing data for remaining features was imputed using the Multiple imputation with chained equations (MICE) was used to impute the remaining missing data in R^42^. Radiomic and numerical clinical features were standardised by centreing on the mean and dividing by the standard deviation (Z-score normalisation)^43^. ComBAT harmonisation was used to correct radiomic features for the batch effect introduced by the use of images from different CT scanners^44^.

To ensure assignment of datasets for training, validation and testing did not lead to bias, the the UK datasets with the most (RMH) and least patients (ICHT) were combined. Cases were then randomly split into training and validation sets with an 80:20 ratio, according to the the binarized outcome. The GSTT dataset was allocated as an external test set. The Lung 1 dataset acted as a second external test set, as described below.

Three models were built for each study endpoint (OS, RFS and recurrence at two years from starting radiotherapy)—one built on radiomic features only, one built on clinical features only, and a combined radiomic-clinical model.

### Statistical analysis

Demographics and clinical parameters are presented with medians and interquartile range for continuous features, and frequencies and percentages for categorical features. Wilcoxon rank sum test (for continuous features) and Fisher’s exact test (for categorical features) were used to summarise comparisons between datasets. The roc.test R package was used to compare ROC curves with 2000 bootstraps. Similar to our previous study^45^, time-to-event data for recurrence, RFS and OS were binarized at two years from the first fraction of radiotherapy for the purpose of classification—cases were labelled “1” if there was recurrence or death within two years, and “0” otherwise. Due to the nature of clinical follow-up, 340 patients (47%) were not seen at or after the two-year endpoint. As simply excluding these patients would bias the datasets, those not known to have had a recurrence or have died within 60 days of the endpoint, or not known to have had a recurrence within 60 days of death, were taken to have no event. Those last seen more than 60 days from the endpoint or date of death were excluded (*n* = 65. 9%).

### Feature handling and modelling

Given the large number of features extracted, feature reduction was required prior to modelling to improve prediction accuracy. We explored a combination of 9 different feature reduction techniques with 11 machine learning algorithms. Feature reduction methods included unsupervised principal component analysis, and 8 supervised approaches applied after first removing highly correlated features (threshold = 0.9) and applying a univariate logistic regression: Boruta, mutual information, recursive feature elimination, correlation-based and multivariate linear penalised methods. A description of algorithms and feature reduction methods are detailed below.

Grid-search was used to identify optimal model hyper-parameters with three repeats of ten-fold cross-validation using the caret package in R. Hyper-parameters of the final selected models are listed in Supplementary Table 3.

The validation set results of each algorithm-feature reduction technique combination were used to create Receiver-Operator Characteristic (ROC) curves and Area Under the Curve (AUC) was calculated. As per our previous study^45^, ensemble prediction models were explored by taking the average of the predictions of the three algorithms with the highest AUC in the validation set for each endpoint being predicted. Where the ensemble model was superior, it was selected as the final model. Otherwise, the single algorithm with the highest AUC was selected as the final model for deployment on the external test set.

### Summary of machine learning algorithms

The models used in this study are supervised classification algorithms. LR—generalised linear model is a generalisation of linear regression to modelling dependencies between predictors and dependent features. Logistic regression is a form of GLM used in this study. It uses the logistic sigmoid function to return a probability value which can then be mapped to two or more separate classes^46^. LASSO and Elastic Net regression can also be used for classification. Ridge is another form of regression where the loss function is modified to minimise the complexity of the model. Here alpha = 0. L-SVM—linear support vector machines plot training samples and assigns a hyperplane (decision boundary) to separate these into classes. The optimal hyperplane is that which maximises the distance between data-points^47^. KNN – K-nearest neighbours assumes that data points that are close to each other are of the same class. It takes a defined number (k) of training samples closest in Euclidian distance to a new point and predicts a class based on these^48^. RF & XGB—Random Forest and Extreme Gradient Boosting Machines are ensemble decision-tree based models. RF uses bagging and feature variability when building each decision tree to create an uncorrelated forest whose overall prediction is more accurate than each individual tree^49^. XGB by contrast takes a boosting approach whereby trees are grown iteratively using information from a previously grown tree, to minimise the error of previous trees^50^. PLS – Partial Least Squares is a multivariate linear regression model which forms linear combinations of features in a supervised manner. It is able to handle datasets with large numbers of features, high collinearity between features and small numbers of observations^51^. NNET—the “nnet” package fits a single-layer feed-forward neural network^52^.

NB—the Naive-Bayes model is based on Bayes theorem and assumes no interdependence between variables^53^.

### Summary of feature reduction techniques

Principal Component Analysis (PCA) is an unsupervised linear transformation technique widely used for feature extraction and dimensionality reduction. PCA identifies directions of maximum variance in high-dimensional data and projects it onto a new subspace with equal or fewer dimensions. Thus, a large set of features is transformed to a smaller one that still retains most of the information in the large set. LASSO (Least Absolute Shrinkage and Selection Operator) and Elastic Net regression are examples of regularisation methods^54^. Here a penalty is applied to the coefficient which multiplies each feature in a linear model and results in less overfitting and improved generalisation. LASSO uses the L1 regularisation penalty to force some coefficients to zero. This eliminates some features leaving a subset of predictors that are thought to be important. Alpha = 1. Elastic Net incorporates penalties from both L1 and L2 (ridge regression) regularisation. Here, Alpha =0·5. The glmnet package was used to perform both LASSO and Elastic-net regression. Pearson, Spearman’s and Kendall’s rank correlation are “filter” feature selection methods which rely only on the characteristics of feature independently of any machine learning model. Pearson’s correlation assumes data is parametric and linear. Spearman and Kendall’s rank are non-parametric and assume a monotonic relationship between variables. Kendall’s rank is preferred to Spearman’s where dataset have a limited number of observations or contain outliers. For our study we used the corr package and specified that the top 25% (8) features be included in the feature sets following Pearson’s, Spearman’s and Kendall’s rank correlation. Univariate LR—apply univariate logistic regression for each feature to the outcome variable and select only those features with a certain *p*-value. We used the glm package with a *p*-value <0.005, adjusted for multiple comparisons with the Benjamini & Hochberg method^46^. RFE—Recursive Feature Elimination is a “wrapper” feature selection method which fits a model and removes the weakest feature until a specified number of features is reached. Cross-validation is used to score different feature subsets and select the best scoring collection of features to identify the optimal number of features. Features are ranked by the model’s feature importance. By recursively eliminating features iteratively the collinearity is reduced^55^. We used the rfeControl package with a random forest model and 10-fold cross validation with 5 repeats. Mutual Information is a filter feature reduction technique that assesses relevance of a subset of features in predicting the target variable compared with redundancy with respect to other variables^56^. Boruta is designed to act as a wrapper around a Random Forest classifier and iteratively removes features which are proved to be less relevant than random probes by statistical testing^57^.

### Integrating clinical data

The combined radiomic-clinical model presented a question of how to best integrate different feature types. After exploring three approaches (Supplementary Material—Integrating clinical data), we decided to use the algorithm-feature reduction technique combination resulting in the best validation set AUC as per the radiomic-only model, and then concatenating clinical features to the input-matrix prior to classification (as described elsewhere in predictive radiomics literature^58–60^).

### Class balancing

The OS and recurrence endpoint datasets were unbalanced (event ratios of 0.34 and 0.36, respectively). Adaptive synthetic sampling (ADASYN)^61^ did not improve validation set AUCs and so the original unbalanced datasets were used (Supplementary Materials—Class balancing).

### Training set size

It has been suggested that predictive power is dependent on training sample size up to a certain point, after which a classifier reaches an efficiency threshold beyond which only marginal or no improvement is seen^62^. We tested robustness of our model development pipeline (Supplementary Material—Training set size) and found that our training set size (*n* = 302) appears sufficient.

### Benchmarking and double external testing for radiomic-OS model

TNM-stage is a known prognostic factor in NSCLC^63^. A logistic regression model based on T and N-stage features (all cases were M0) was developed to benchmark our prediction models against. As above, the Caret package was used with hyper-parameter optimisation performed via grid-search with three repeats of ten-fold cross-validation. AUC was calculated.

For the Radiomic-only OS model, the publicly available Lung 1 dataset was used as a second external test set. It was not possible to test our other models with this dataset owing to lack of matching clinical features and recurrence outcomes.

### Risk-groups

The Youden Index of the validation set ROC curve for each final model was used to separate groups into high (event occurs within two years of first fraction of radiotherapy) or low (event does not occur within two years of first fraction of radiotherapy) risk groups. The full time to event data were used to create Kaplan Meier curves which demonstrated difference in OS/RFS/recurrence between groups. The log-rank test (significance level = 0.05) was used to determine difference between survival curves. The external test set was used to assess performance of the risk models. Analyses were carried out in R 3.5.1.

### Lymph node involvement

Radiomic models tend to focus on a single VOI around the primary tumour, however afflicted nodal regions may also harbour valuable prognostic information. We therefore explored how radiomics from nodal regions may perform either in comparison or combination to that of the primary tumour in a subset of 127 patients with separately contoured primary tumour and nodal GTVs. These were divided into training (80%) and validation (20%) sets, stratified by outcome. Image pre-processing and feature extraction methods were as described above. Highly correlated features were removed (threshold 0.9). Utilising univariate logistic regression for feature reduction (as in the main study) failed in this sub-study and an XGBoost model was therefore applied instead. Three models were compared: one built on features from the primary, one built on features from the largest LN region, and a model built on these features combined.

### Quality assurance

For quality assurance, the radiomics quality score^64^ was calculated and TRIPOD recommendations^65^ and IBSI reporting guidelines^66^ were followed (Supplementary Materials).

### Reporting summary

Further information on research design is available in the Nature Research Reporting Summary linked to this article.

## Supplementary information

Supplemental material

REPORTING SUMMARY

Supplemental Material

## Data Availability

Due to confidentiality, data collected for the study are not publicly available for download, however the corresponding authors can be contacted for academic inquiries. The radiomics data generated in this study have been deposited into the Mendeley database under the accession code 10.17632/4fh598c8w2.1^67^.

## Code availability

Tools for machine learning are indicated in the methods section. The R script used to reproduce the key findings and generate figures have been deposited into the Mendeley database under the accession code 10.17632/4fh598c8w2.1^67^.
