# Author Correction: Gross tumour volume radiomics for prognostication of recurrence & death following radical radiotherapy for NSCLC

**DOI:** 10.1038/s41698-022-00332-1

**Published:** 2022-11-23

**Authors:** Sumeet Hindocha, Thomas G. Charlton, Kristofer Linton-Reid, Benjamin Hunter, Charleen Chan, Merina Ahmed, Emily J. Greenlay, Matthew Orton, Catey Bunce, Jason Lunn, Simon J. Doran, Shahreen Ahmad, Fiona McDonald, Imogen Locke, Danielle Power, Matthew Blackledge, Richard W. Lee, Eric O. Aboagye

**Affiliations:** 1 Lung Unit, The Royal Marsden NHS Foundation Trust, London, UK; 2 AI for Healthcare Centre for Doctoral Training, Imperial College London, London, UK; 3 Institute of Cancer Research NIHR Biomedical Research Centre, London, UK; 4 Cancer Imaging Centre, Department of Surgery & Cancer, Imperial College London, London, UK; 5 Guy’s Cancer Centre, Guy’s and St Thomas’ NHS Foundation Trust, London, UK; 6 Lung Unit, The Royal Marsden NHS Foundation Trust, Sutton, UK; 7 Clinical Trials Unit, Royal Marsden NHS Foundation Trust, Sutton, UK; 8 Artificial Intelligence Imaging Hub, Royal Marsden NHS Foundation Trust, Sutton, UK; 9 Department of Clinical Oncology, Charing Cross Hospital, London, UK; 10 Radiotherapy and Imaging, Institute of Cancer Research, London, UK; 11 Early Diagnosis and Detection Team, Institute of Cancer Research, London, UK

**Keywords:** Non-small-cell lung cancer, Cancer imaging, Cancer models, Radiotherapy, Tumour biomarkers

Correction to: *npj Precision Oncology* 10.1038/s41698-022-00322-3, published online 27 October 2022.

“In this article the affiliation details for Eric O. Aboagye were incorrectly given as ‘Institute of Cancer Research NIHR Biomedical Research Centre, London, UK’ but should have been ‘Cancer Imaging Centre, Department of Surgery & Cancer, Imperial College London, London, UK’. The original article has been corrected.”

