# Supplementary material for: Gross tumour volume radiomics for prognostication of recurrence & death following radical radiotherapy for NSCLC
Source: NPJ Precis Oncol. 2022 Oct 27;6:77. doi: 10.1038/s41698-022-00322-3 (PMC9613990; doi:10.1038/s41698-022-00322-3)
Supplement: Supplementary file 1 — Supplemental material [file 41698_2022_322_MOESM1_ESM.pdf]

Supplementary Material – Gross Tumour Volume CT Radiomics for Prognostication of Recurrence & Death following Curative-Intent Radiotherapy for Non-Small Cell Lung Cancer:

Table of Contents:

Integrating clinical data & supplementary table 1
Class balancing & supplementary table 2
Training set size & supplementary figure 1
Correlation of radiomic with clinical features & supplementary figure 2
Radiomics Quality Score
TRIPOD recommendations
IBSI reporting guidelines
Supplementary Table 3 – Hyper-parameters
Supplementary Table 4 – The full list of radiomic features used in the study
Supplementary Table 5 – Radiomic features surviving feature reduction for two-year OS and RFS
Supplementary Table 6 – The 15 Radiomic features most contributing to predicting recurrence at two years post treatment
Supplementary Table 7 – Classification metrics
Supplementary Table 8 – Brier Scores
Supplementary Figure 1 – Calibration Curves
Supplementary Material References

Integrating clinical data

With radiomic studies increasingly incorporating clinical data, this presents a question as to the most appropriate method for combing the different data types. We explored three approaches evaluated by validation set AUC:

1. Using the algorithm-feature reduction technique combination with the best validation set AUC as per the radiomic-only model, then concatenating clinical features to the input-matrix prior to classification
2. Using the best algorithm-feature reduction technique combination for radiomic features and the best combination for the clinical features, then ensembling these models by averaging their predictions
3. Or by ensembling these models with a Partial Least Squares (PLS) classifier.

Approach 1 resulted in the highest validation set AUC for OS (supplementary table 1) and was applied for the Combined models for RFS and recurrence also. The method resulting in the highest AUC has been utilised elsewhere as described in the main paper, however it's important to consider that AUC alone may not be the most appropriate evaluation metric and there is a need for development of more methodology focusing on the combination of radiomic and clinical data.

**Approach**	**1**	**2**	**3**
**OS Validation set AUC**	0·702	0·6736	0·6784
**OS Validation set AUC CI**	0·5831-0·8217	0·5497-0·7975	0·5559-0·8009

Supplementary table 1. Exploring 3 different approaches to combine radiomic and clinical features. Results are in the form of validation set AUC and 95% CI for the OS endpoint. Approach 1 resulted in the highest validation set AUC and was selected as the approach to combine radiomic and clinical features for the Combined models for RFS and recurrence endpoints.

Class balancing

The OS and recurrence endpoint datasets were unbalanced (event ratios of 0.34 and 0.36, respectively). To investigate whether class balancing improved results, we applied adaptive synthetic sampling ADASYN prior to modelling and compared validation set AUCs with the original unbalanced datasets[15]. When averaging across all machine learning algorithms for Spearman (OS) and PCA (Recurrence), applying ADASYN[15] to balance outcomes in the training set did not result in superior validation set results (supplementary table 2). The original unbalanced training sets were therefore used for modelling.

**Endpoint**	**Recurrence**		**OS**	
**Selected feature reduction technique**	**PCA**	**PCA (with ADASYN)**	**Spearman**	**Spearman (with ADASYN)**
**XGB**	0·623	0·573	0·667	0·601
**RF**	0·595	0·597	0·671	0·636
**NB**	0·546	0·511	0·700	0·700
**PLS**	0·661	0·649	0·712	0·716
**NNET**	0·530	0·545	0·627	0·604
**L-SVM**	0·532	0·589	0·550	0·620
**LR**	0·632	0·629	0·654	0·656
**KNN**	0·650	0·645	0·682	0·616
**Ridge**	0·634	0·629	0·708	0·715
**LASSO**	0·639	0·613	0·682	0·690
**ENET**	0·641	0·610	0·685	0·689
**Mean**	0·607	0·599	0·667	0·658

Supplementary table 2. Validation set AUCs for PCA (predicting recurrence) and Spearman (predicting OS) feature reduction techniques, with and without applying ADASYN to balance outcomes in the training set. Averaging across all machine algorithms used, ADASYN did not result in superior AUC values and therefore the original training sets were used for modelling.

Training set size
Training set size is an important consideration when developing radiomic-based prediction models. Data availability can often be limited in clinical studies especially when this is performed retrospectively from electronic health records. It has been suggested however that predictive power is dependent on training sample size up to a certain point, after which a classifier reaches an efficiency threshold beyond which only marginal or no improvement is seen[16]. To test robustness of our model development pipeline, we compared validation set AUCs with varying training set sizes, using the RFS endpoint with Recursive Feature Elimination and Naïve-Bayes as a random algorithm-feature reduction technique combination example. Supplementary figure 1 shows results of our experiments with varying training set size. Stability in validation set AUC is reached with training set >180 cases, thus demonstrating that our n=302 appears sufficient. At approximately 125 cases AUC drops by ~3%, a change which could be observed by chance as different cases enter the training set at random. As training set size increases, the overall trend of increasing AUC emerges. This is also seen in[17].

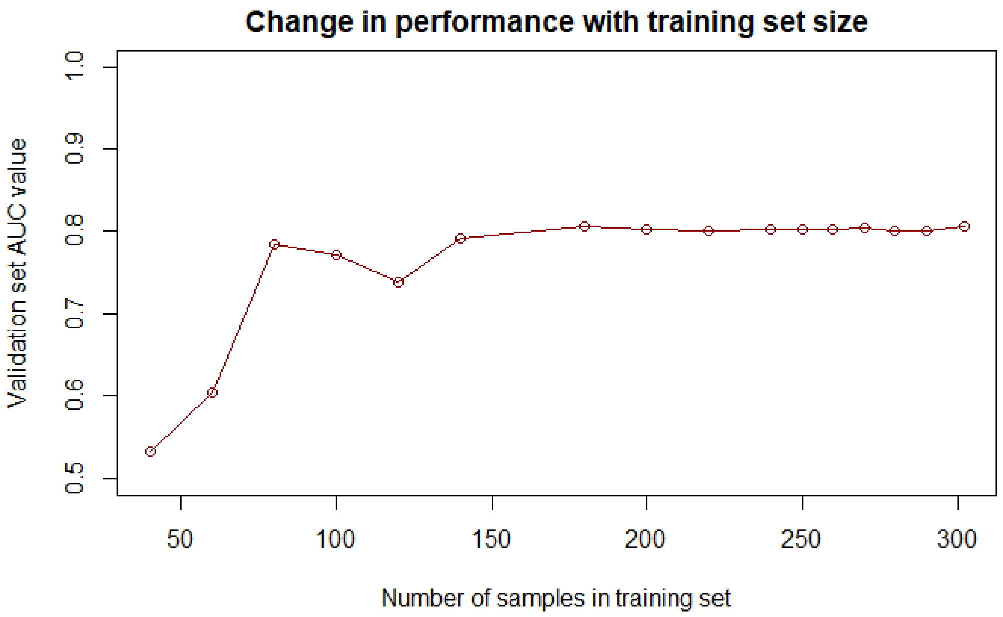

Supplementary figure 1. Validation set AUC values with varying training set size. Values stabilise once training set size reaches beyond 180 cases.

Correlation of Radiomic with Clinical Features
We explored the association between the radiomic features used in each model (OS, RFS, recurrence, as detailed in Supplementary Tables 5 & 6 below), with the clinical features. Correlation heatmaps are shown in Supplementary Figure 2. For OS, there was a strong association between T1-stage and NGLDM_Coarseness, a texture feature that measures the level of spatial rate of change in intensity. Size of primary tumour (which correlates with T-stage) was also strongly associated with other texture features including Grey-level run-length matrix (GLRLM), Gray Level Size Zone (GLSZM) and Gray Level Co-occurrence Matrix (GLCM) features. Interestingly, a weak association between GLSZM is also noted with nodal avidity and nodal SUV, though this may be due to larger tumours more likely having advanced nodal involvement. For predicting RFS, size of primary tumour was strongly associated with the same texture features as for OS, as well as the median gray level intensity within the ROI (FOS_lmedian_LLL). T1-stage disease was associated with the square-root of the mean of all the squared intensity values (FOS_RMS). With recurrence, the strongest associations were between T1-stage and GLSZM Zone Percentage, which measures the coarseness of the texture by taking the ratio of number of zones and number of voxels in the ROI, and GLRLM.

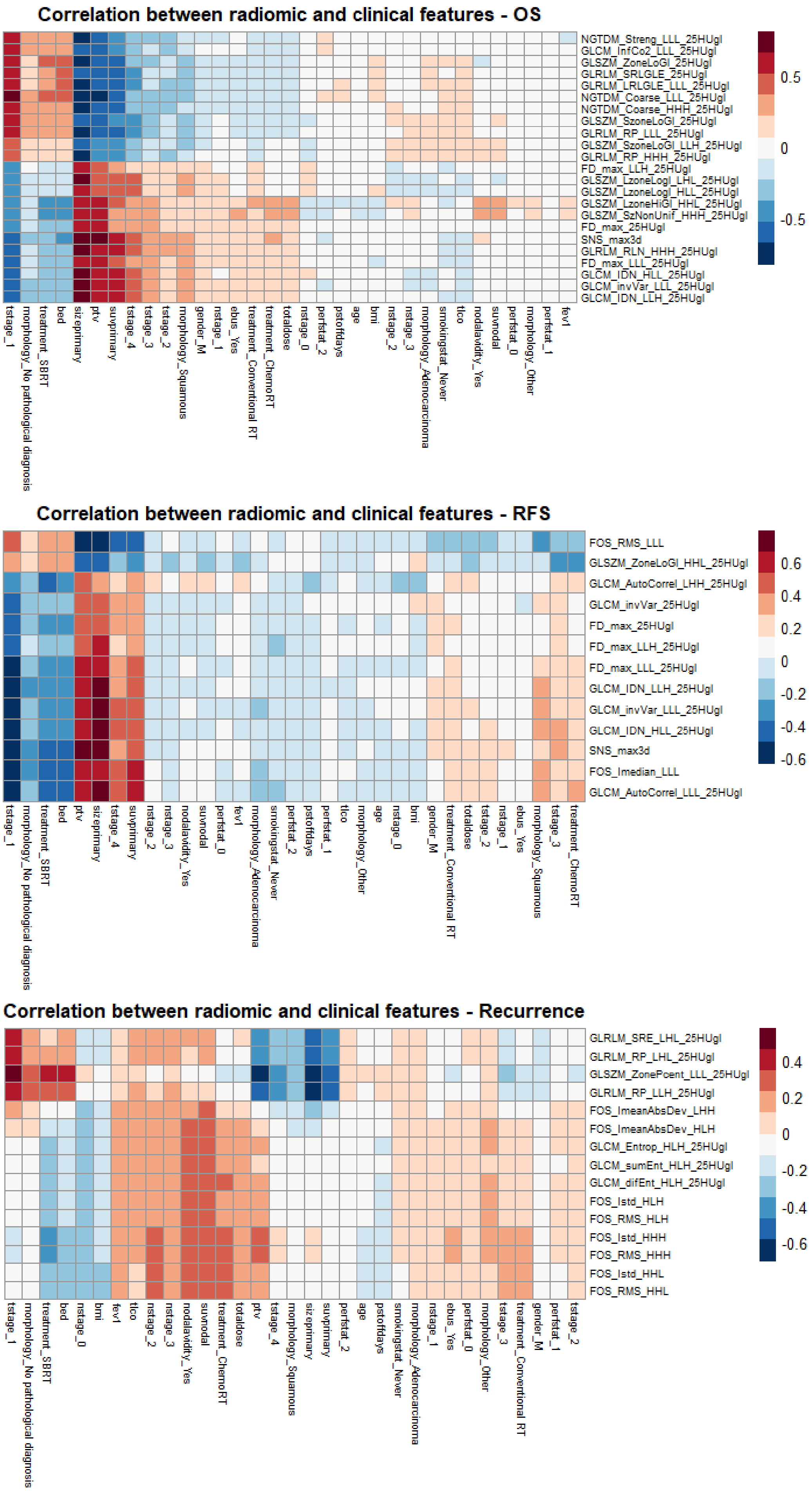

Supplementary Figure 2. Correlation heatmaps of the radiomic features used for modelling each endpoint, against clinical features. Positive associations are in red and negative in blue.

The Radiomics Quality Score[18] for our study is tabulated below. Our study scores comparatively high according to two studies evaluating studies against the radiomics quality score[19,20].

Criteria	Points	Our Score
1. Image protocol quality – well-documented image protocols (for example, contrast, slice thickness, energy, etc.) and/or usage of public image protocols allow reproducibility/replicability	+ 1 (if protocols are well-documented) + 1 (if public protocol is used)	0
2. Multiple segmentations – possible actions are: segmentation by different physicians/algorithms/software, perturbing segmentations by (random) noise, segmentation at different breathing cycles. Analyse feature robustness to segmentation variabilities	+ 1	0
3. Phantom study on all scanners – detect inter-scanner differences and vendor-dependent features. Analyse feature robustness to these sources of variability	+ 1	0
4. Imaging at multiple time points – collect images of individuals at additional time points. Analyse feature robustness to temporal variabilities (for example, organ movement, organ expansion/shrinkage)	+ 1	0
5. Feature reduction or adjustment for multiple testing – decreases the risk of overfitting. Overfitting is inevitable if the number of features exceeds the number of samples. Consider feature robustness when selecting features	− 3 (if neither measure is implemented) + 3 (if either measure is implemented)	3
6. Multivariable analysis with non-radiomics features (for example, EGFR mutation) – is expected to provide a more holistic model. Permits correlating/inferencing between radiomics and non-radiomics features	+ 1	1
7. Detect and discuss biological correlates – demonstration of phenotypic differences (possibly associated with underlying gene–protein expression patterns) deepens understanding of radiomics and biology	+ 1	1
8. Cut-off analyses – determine risk groups by either the median, a previously published cut-off or report a continuous risk variable. Reduces the risk of reporting overly optimistic results	+ 1	1
9. Discrimination statistics – report discrimination statistics (for example, C-statistic, ROC curve, AUC) and their statistical significance (for example, p-values, confidence intervals). One can also apply resampling method (for example, bootstrapping, cross-validation)	+ 1 (if a discrimination statistic and its statistical significance are reported) + 1 (if a resampling method technique is also applied)	2
10. Calibration statistics – report calibration statistics (for example, Calibration-in-the-large/slope, calibration plots) and their statistical significance (for example, *P*-values, confidence intervals). One can also apply resampling method (for example, bootstrapping, cross-validation)	+ 1 (if a calibration statistic and its statistical significance are reported) + 1 (if a resampling method technique is also applied)	2
11. Prospective study registered in a trial database – provides the highest level of evidence supporting the clinical validity and usefulness of the radiomics biomarker	+ 7 (for prospective validation of a radiomics signature in an appropriate trial)	0
12. Validation – the validation is performed without retraining and without adaptation of the cut-off value, provides crucial information with regard to credible clinical performance	- 5 (if validation is missing) + 2 (if validation is based on a dataset from the same institute) + 3 (if validation is based on a dataset from another institute) + 4 (if validation is based on two datasets from two distinct institutes) + 4 (if the study validates a previously published signature) + 5 (if validation is based on three or more datasets from distinct institutes) *Datasets should be of comparable size and should have at least 10 events per model feature	4
13. Comparison to 'gold standard' – assess the extent to which the model agrees with/is superior to the current 'gold standard' method (for example, TNM-staging for survival prediction). This comparison shows the added value of radiomics	+ 2	2
14. Potential clinical utility – report on the current and potential application of the model in a clinical setting (for example, decision curve analysis).	+ 2	2
15. Cost-effectiveness analysis - report on the cost-effectiveness of the clinical application (for example, QALYs generated)	+ 1	0
16. Open science and data - make code and data publicly available. Open science facilitates knowledge transfer and reproducibility of the study	+ 1 (if scans are open source) + 1 (if region of interest segmentations are open source) + 1 (if code is open source) + 1 (if radiomics features are calculated on a set of representative ROIs and the calculated features and representative ROIs are open source)	0
Total points (36 = 100%)	36	18 (50%)

TRIPOD recommendations. An "X" indicates if the objective is addressed in the study:

Section/Topic	ι	Checklist Item	Page
**Title and abstract**		**CT Radiomics Models for Prognostication and Risk-Stratification of Recurrence & Death after Curative-Intent Radiotherapy for Non-Small Cell Lung Cancer**	

Title	1	Identify the study as developing and/or validating a multivariable prediction model, the target population, and the outcome to be predicted.	1
Abstract	2	Provide a summary of objectives, study design, setting, participants, sample size, predictors, outcome, statistical analysis, results, and conclusions.	1
**Introduction**			
Background and objectives	3a	Explain the medical context (including whether diagnostic or prognostic) and rationale for developing or validating the multivariable prediction model, including references to existing models.	3
	3b	Specify the objectives, including whether the study describes the development or validation of the model or both.	3
**Methods**			
Source of data	4a	Describe the study design or source of data (e.g., randomized trial, cohort, or registry data), separately for the development and validation data sets, if applicable.	4
	4b	Specify the key study dates, including start of accrual; end of accrual; and, if applicable, end of follow-up.	4/SM
Participants	5a	Specify key elements of the study setting (e.g., primary care, secondary care, general population) including number and location of centres.	4/SM
	5b	Describe eligibility criteria for participants.	4/SM
	5c	Give details of treatments received, if relevant.	4/SM
Outcome	6a	Clearly define the outcome that is predicted by the prediction model, including how and when assessed.	4
	6b	Report any actions to blind assessment of the outcome to be predicted.	
Predictors	7a	Clearly define all predictors used in developing or validating the multivariable prediction model, including how and when they were measured.	SM
	7b	Report any actions to blind assessment of predictors for the outcome and other predictors.	
Sample size	8	Explain how the study size was arrived at.	
Missing data	9	Describe how missing data were handled (e.g., complete-case analysis, single imputation, multiple imputation) with details of any imputation method.	SM
Statistical analysis methods	10a	Describe how predictors were handled in the analyses.	4
	10b	Specify type of model, all model-building procedures (including any predictor selection), and method for internal validation.	4
	10d	Specify all measures used to assess model performance and, if relevant, to compare multiple models.	5
Risk groups	11	Provide details on how risk groups were created, if done.	6
**Results**			
Participants	13a	Describe the flow of participants through the study, including the number of participants with and without the outcome and, if applicable, a summary of the follow-up time. A diagram may be helpful.	4
	13b	Describe the characteristics of the participants (basic demographics, clinical features, available predictors), including the number of participants with missing data for predictors and outcome.	6
Model development	14a	Specify the number of participants and outcome events in each analysis.	6
	14b	If done, report the unadjusted association between each candidate predictor and outcome.	
Model specification	15a	Present the full prediction model to allow predictions for individuals (i.e., all regression coefficients, and model intercept or baseline survival at a given time point).	
	15b	Explain how to the use the prediction model.	
Model performance	16	Report performance measures (with CIs) for the prediction model.	8
**Discussion**			
Limitations	18	Discuss any limitations of the study (such as nonrepresentative sample, few events per predictor, missing data).	16
Interpretation	19b	Give an overall interpretation of the results, considering objectives, limitations, and results from similar studies, and other relevant evidence.	15
Implications	20	Discuss the potential clinical use of the model and implications for future research.	16
**Other information**			
Supplementary information	21	Provide information about the availability of supplementary resources, such as study protocol, Web calculator, and data sets.	17/SM
Funding	22	Give the source of funding and the role of the funders for the present study.	6
SM = Supplementary Material			

IBSI reporting guidelines: "Y" indicates which sections are reported in the manuscript. Sections not relating to CT imaging are marked as not applicable ("NA").

IBSI Reporting Guidelines				
Patient				
**Topic**	**Modality**	**Item**	**Description**	**Included in manuscript**
Region of interest [1]		1	Describe the region of interest that is being imaged.	Y
Patient preparation		2a	Describe specific instructions given to patients prior to image acquisition, e.g. fasting prior to imaging.	
		2b	Describe administration of drugs to the patient prior to image acquisition, e.g. muscle relaxants.	
		2c	Describe the use of specific equipment for patient comfort during scanning, e.g. ear plugs.	
Radioactive tracer	PET, SPECT	3a	Describe which radioactive tracer was administered to the patient, e.g. 18F-FDG.	NA
	PET, SPECT	3b	Describe the administration method.	NA
	PET, SPECT	3c	Describe the injected activity of the radioactive tracer at administration.	NA
	PET, SPECT	3d	Describe the uptake time prior to image acquisition.	NA
	PET, SPECT	3e	Describe how competing substance levels were controlled. [2]	NA
Contrast agent		4a	Describe which contrast agent was administered to the patient.	
		4b	Describe the administration method.	
		4c	Describe the injected quantity of contrast agent.	
		4d	Describe the uptake time prior to image acquisition.	
		4e	Describe how competing substance levels were controlled.	
Comorbidities		5	Describe if the patients have comorbidities that affect imaging. [3]	
Acquisition				
**Topic**	**Modality**	**Item**	**Description**	**Included**
Acquisition protocol		6	Describe whether a standard imaging protocol was used, and where its description may be found.	
Scanner type		7	Describe the scanner type(s) and vendor(s) used in the study.	
Imaging modality		8	Clearly state the imaging modality that was used in the study, e.g. CT, MRI.	
Static/dynamic scans		9a	State if the scans were static or dynamic.	
	Dynamic scans	9b	Describe the acquisition time per time frame.	
	Dynamic scans	9c	Describe any temporal modelling technique that was used.	
Scanner calibration		10	Describe how and when the scanner was calibrated.	
Patient instructions		11	Describe specific instructions given to the patient during acquisition, e.g. breath holding.	
Anatomical motion correction		12	Describe the method used to minimise the effect of anatomical motion.	
Scan duration		13	Describe the duration of the complete scan or the time per bed position.	
Tube voltage	CT	14	Describe the peak kilo voltage output of the X-ray source.	
Tube current	CT	15	Describe the tube current in mA.	

Time-of-flight	PET	16	State if scanner time-of-flight capabilities are used during acquisition.	NA
RF coil	MRI	17	Describe what kind RF coil used for acquisition, incl. vendor.	NA
Scanning sequence	MRI	18a	Describe which scanning sequence was acquired.	NA
	MRI	18b	Describe which sequence variant was acquired.	NA
	MRI	18c	Describe which scan options apply to the current sequence, e.g. flow compensation, cardiac gating.	NA
Repetition time	MRI	19	Describe the time in ms between subsequent pulse sequences.	NA
Echo time	MRI	20	Describe the echo time in ms.	NA
Echo train length	MRI	21	Describe the number of lines in k-space that are acquired per excitation pulse.	NA
Inversion time	MRI	22	Describe the time in ms between the middle of the inverting RF pulse to the middle of the excitation pulse.	NA
Flip angle	MRI	23	Describe the flip angle produced by the RF pulses.	NA
Acquisition type	MRI	24	Describe the acquisition type of the MRI scan, e.g. 3D.	NA
k-space traversal	MRI	25	Describe the acquisition trajectory of the k-space.	NA
Number of averages/ excitations	MRI	26	Describe the number of times each point in k-space is sampled.	NA
Magnetic field strength	MRI	27	Describe the nominal strength of the MR magnetic field.	NA
Reconstruction				
**Topic**	**Modality**	**Item**	**Description**	**Included**
In-plane resolution		28	Describe the distance between pixels, or alternatively the field of view and matrix size.	
Image slice thickness		29	Describe the slice thickness.	Y
Image slice spacing		30	Describe the distance between image slices. [6]	
Convolution kernel	CT	31a	Describe the convolution kernel used to reconstruct the image.	
	CT	31b	Describe settings pertaining to iterative reconstruction algorithms.	
Exposure	CT	31c	Describe the exposure (in mAs) in slices containing the region of interest.	
Reconstruction method	PET	32a	Describe which reconstruction method was used, e.g. 3D OSEM.	NA
	PET	32b	Describe the number of iterations for iterative reconstruction.	NA
	PET	32c	Describe the number of subsets for iterative reconstruction.	NA
Point spread function modelling	PET	33	Describe if and how point-spread function modelling was performed.	NA
Image corrections	PET	34a	Describe if and how attenuation correction was performed.	NA
	PET	34b	Describe if and how other forms of correction were performed, e.g. scatter correction, randoms correction, dead time correction etc.	NA
Reconstruction method	MRI	35a	Describe the reconstruction method used to reconstruct the image from the k-space information.	NA
	MRI	35b	Describe any artifact suppression methods used during reconstruction to suppress artifacts due to undersampling of k-space.	NA

Diffusion-weigh ted imaging	DWI-MRI	36	Describe the b-values used for diffusion-weigh ting.	NA
		Image registration		
**Topic**	**Modality**	**Item**	**Description**	**Included**
Registration method		37	Describe the method used to register multi-modality imaging.	NA
		Image processing		
		Data conversion		
**Topic**	**Modality**	**Item**	**Description**	**Included**
SUV normalisation	PET	38	Describe which standardised uptake value (SUV) normalisation method is used.	NA
ADC computation	DWI-MRI	39	Describe how apparent diffusion coefficient (ADC) values were calculated.	NA
Other data conversions		40	Describe any other conversions that are performed to generate e.g. perfusion maps.	NA
		Post-acquisition processsing		
**Topic**	**Modality**	**Item**	**Description**	**Included**
Anti-aliasing		41	Describe the method used to deal with anti-aliasing when down-sampling during interpolation.	Y
Noise suppression		42	Describe methods used to suppress image noise.	
Post-reconstruc tion smoothing filter	PET	43	Describe the width of the Gaussian filter (FWHM) to spatially smooth intensities.	NA
Skull stripping	MRI (brain)	44	Describe method used to perform skull stripping.	NA
Non-uniformity correction [7]	MRI	45	Describe the method and settings used to perform non-uniformity correction.	NA
Intensity normalisation		46	Describe the method and settings used to normalise intensity distributions within a patient or patient cohort.	
Other post-acquisitio n processing methods		47	Describe any other methods that were used to process the image and are not mentioned separately in this list.	Y
		Segmentation		
**Topic**	**Modality**	**Item**	**Description**	**Included**
Segmentation method		48a	Describe how regions of interest were segmented, e.g. manually.	Y
		48b	Describe the number of experts, their expertise and consensus strategies for manual delineation.	Y
		48c	Describe methods and settings used for semi-automatic and fully automatic segmentation.	NA
		48d	Describe which image was used to define segmentation in case of multi-modality imaging.	NA
Conversion to mask		49	Describe the method used to convert polygonal or mesh-based segmentations to a voxel-based mask.	NA
		Image interpolation		
**Topic**	**Modality**	**Item**	**Description**	**Included**
Interpolation method		50a	Describe which interpolation algorithm was used to interpolate the image.	Y
		50b	Describe how the position of the interpolation grid was defined, e.g. align by center.	Y
		50c	Describe how the dimensions of the interpolation grid were defined, e.g. rounded to nearest integer.	Y
		50d	Describe how extrapolation beyond the original image was handled.	NA

Voxel dimensions		51	Describe the size of the interpolated voxels.	Y
Intensity rounding	CT	52	Describe how fractional Hounsfield Units are rounded to integer values after interpolation.	Y
ROI interpolation				
**Topic**	**Modality**	**Item**	**Description**	**Included**
Interpolation method		53	Describe which interpolation algorithm was used to interpolate the region of interest mask.	Y
Partially masked voxels		54	Describe how partially masked voxels after interpolation are handled.	Y
Re-segmentation				
**Topic**	**Modality**	**Item**	**Description**	**Included**
Re-segmentation methods		55	Describe which methods and settings are used to re-segment the ROI intensity mask.	NA
Discretisation				
**Topic**	**Modality**	**Item**	**Description**	**Included**
Discretisation method [8]		56a	Describe the method used to discretise image intensities.	Y
		56b	Describe the number of bins (FBN) or the bin size (FBS) used for discretisation.	Y
		56c	Describe the lowest intensity in the first bin for FBS discretisation.	
Image transformation				
**Topic**	**Modality**	**Item**	**Description**	**Included**
Image filter [10]		57	Describe the methods and settings used to filter images, e.g. Laplacian-of-Ga ussian.	Y
Image biomarker computation				
**Topic**	**Modality**	**Item**	**Description**	**Included**
Biomarker set		58	Describe which set of image biomarkers is computed and refer to their definitions or provide these.	Y
IBSI compliance		59	State if the software used to extract the set of image biomarkers is compliant with the IBSI benchmarks.	Y
Robustness		60	Describe how robustness of the image biomarkers was assessed, e.g. test-retest analysis.	Y
Software availability		61	Describe which software and version was used to compute image biomarkers.	Y
Image biomarker computation - texture parameters				
**Topic**	**Modality**	**Item**	**Description**	**Included**
Texture matrix aggregation		62	Define how texture-matrix based biomarkers were computed from underlying texture matrices.	Y
Distance weighting		63	Define how CM, RLM, NGTDM and NGLDM weight distances, e.g. no weighting.	
CM symmetry		64	Define whether symmetric or asymmetric co-occurrence matrices were computed.	
CM distance		65	Define the (Chebyshev) distance at which co-occurrence of intensities is determined, e.g. 1.	
SZM linkage distance		66	Define the distance and distance norm for which voxels with the same intensity are considered to belong to the same zone for the purpose of constructing an SZM, e.g. Chebyshev distance of 1.	
DZM linkage distance		67	Define the distance and distance norm for which voxels with the same intensity are considered to belong to the same zone for the purpose of constructing a DZM, e.g. Chebyshev distance of 1.	
DZM zone distance norm		68	Define the distance norm for determining the distance of zones to the border of the ROI, e.g. Manhattan distance.	
NGTDM distance		69	Define the neighbourhood distance and distance norm for the NGTDM, e.g. Chebyshev distance of 1.	

NGLDM distance		70	Define the neighbourhood distance and distance norm for the NGLDM, e.g. Chebyshev distance of 1.	
NGLDM coarseness		71	Define the coarseness parameter for the NGLDM, e.g. 0.	
Machine learning and radiomics analysis				
**Topic**	**Modality**	**Item**	**Description**	**Included**
Diagnostic and prognostic modelling		72	See the TRIPOD guidelines for reporting on diagnostic and prognostic modelling.	Y
Comparison with known factors		73	Describe where performance of radiomics models is compared with known (clinical) factors.	Y
Multicollineari ty		74	Describe where the multicollineari ty between image biomarkers in the signature is assessed.	Y
Model availability		75	Describe where radiomics models with the necessary pre-processing information may be found.	Y
Data availability		76	Describe where imaging data and relevant meta-data used in the study may be found.	Y

Supplementary Table 3: Hyperparameters used for the Radiomic and Combined models. Algorithms used were PLS (predicting OS and RFS) and KNN, PLS and E-Net for the ensemble model (predicting recurrence).

Endpoint	Model	Algorithm	Library	Hyperparameter
OS	Radiomic	PLS	*pls*	ncomp = 1
	Combined	PLS	*pls*	ncomp = 1
RFS	Radiomic	PLS	*pls*	ncomp = 1
	Combined	PLS	*pls*	ncomp = 1
Recurrence	Radiomic	KNN	*knn*	k = 27
		PLS	*pls*	ncomp = 1
		E-Net	*glmnet*	alpha = 0·5, lambda = 0·0199951
	Combined	KNN	*knn*	k = 29
		PLS	*pls*	ncomp = 1
		E-Net	*glmnet*	alpha = 0·5, lambda = 0·03029972

Supplementary Table 4: The full list of radiomic features used in the study.

Technique (Total number)	Features
First order statistics (FOS)<br>(15)	1. Coefficient of Variation<br>2. Mean<br>3. Median<br>4. Mode<br>5. Standard Deviation<br>6. Minimum<br>7. Maximum<br>8. Range<br>9. Skewness<br>10. Kurtosis<br>11. Mean Absolute Deviation<br>12. Root mean square<br>13. Area under the Curve<br>14. Entropy<br>15. Energy

Grey-level co-occurrence matrix (GLCM)<br>(23)	1. Variance<br>2. Correlation<br>3. Information Measure of Correlation 1)<br>4. Information Measure of Correlation 2)<br>5. Cluster Shade<br>6. Cluster Prominence<br>7. Angular Second Moment<br>8. Maximum Probability<br>9. Entropy<br>10. Contrast<br>11. Dissimilarity<br>12. Homogeneity<br>13. Sum Average<br>14. Sum Variance<br>15. Sum Entropy<br>16. Difference in Variance<br>17. Difference entropy<br>18. Autocorrelation<br>19. Cluster Tendency<br>20. Homogeneity 1<br>21. Inverse Difference Moment Normalised<br>22. Inverse Difference Normalised<br>23. Inverse Variance
Grey-level size zone matrix (GLSZM)<br>(13)	1. Small Zone Emphasis<br>2. Large Zone Emphasis<br>3. Grey-level Non Uniformity<br>4. Size zone Non uniformity<br>5. Zone Percentage<br>6. Zone Low grey-level Emphasis<br>7. Zone Low grey-level Emphasis<br>8. Small Zone Low grey level Zone Emphasis<br>9. Small Zone High grey level Zone Emphasis<br>10. Large Zone Low grey level Emphasis<br>11. Large Zone High grey level Emphasis<br>12. Grey Level Variance<br>13. Size-Zone Variance
Neighborhood grey- tone difference matrix (NGTDM)<br>(5)	1. Coarseness<br>2. Contrast<br>3. Busyness<br>4. Complex<br>5. Strength
Size and Shape (SNS)<br>(8)	1. Volume<br>2. Area<br>3. Surface to volume ratio<br>4. Sphericity<br>5. Spherical disproportion<br>6. Compactness 1<br>7. Compactness 2<br>8. Maximum 3d diameter
Grey-Level Run Length Matrix (GLRLM)<br>(11)	1. Short Run Emphasis<br>2. Long Run Emphasis<br>3. Grey-Level Non-Uniformity<br>4. Run Length Non-Uniformity<br>5. Run Percentage<br>6. Low Grey-Level Run Emphasis<br>7. High Grey Level Run Emphasis<br>8. Short Run Low Grey Level Emphasis<br>9. Short Run High Grey Level Emphasis<br>10. Long Run Low Grey Level Emphasis<br>11. Long Run High Grey Level Emphasis
Fractal Dimension (FD)<br>(6)	1. Mean<br>2. Standard deviation<br>3. Variance<br>4. Lacunarity<br>5. Maximum<br>6. Minimum
Wavelet transformations x8	As above but with 8 filters

Supplementary Table 5: The list of radiomic features surviving feature reduction for OS and RFS. Feature reduction techniques used are Spearman and Pearson's rank correlation, selecting the top 20% of features remaining after first removing highly correlated features and applying a univariate logistic regression. SNS = size and shape features, GLSZM = Grey-level size zone matrix, FOS = first order statistics, GLRLM = Grey-Level Run Length Matrix, FD = fractal dimensions, NGTDM = Neighbourhood grey- tone difference matrix, GLCM = Grey-level co-occurrence matrix

OS & Spearman's rank correlation	RFS & Pearson's rank correlation
1. SNS_max3d	1. SNS_max3d
2. GLSZM_ZoneLoGl_25HUgl	2. FD_max_25HUgl
3. GLSZM_SzoneLoGl_25HUgl	3. GLCM_invVar_25HUgl
4. GLRLM_SRLGLE_25HUgl	4. FOS_Imedian_LLL
5. FD_max_25HUgl	5. FOS_RMS_LLL
6. NGTDM_Coarse_LLL_25HUgl	6. FD_max_LLL_25HUgl
7. NGTDM_Streng_LLL_25HUgl	7. GLCM_AutoCorrel_LLL_25HUgl
8. GLRLM_RP_LLL_25HUgl	8. GLCM_invVar_LLL_25HUgl
9. GLRLM_LRLGLE_LLL_25HUgl	9. FD_max_LLH_25HUgl
10. FD_max_LLL_25HUgl	10. GLCM_IDN_LLH_25HUgl
11. GLCM_InfCo2_LLL_25HUgl	11. GLCM_AutoCorrel_LHH_25HUgl
12. GLCM_invVar_LLL_25HUgl	12. GLCM_IDN_HLL_25HUgl
13. GLSZM_SzoneLoGl_LLH_25HUgl	13. GLSZM_ZoneLoGl_HHL_25HUgl
14. FD_max_LLH_25HUgl	
15. GLCM_IDN_LLH_25HUgl	
16. GLSZM_LzoneLogl_LHL_25HUgl	
17. GLSZM_LzoneLogl_HLL_25HUgl	
18. GLCM_IDN_HLL_25HUgl	
19. GLSZM_LzoneHiGl_HHL_25HUgl	
20. GLSZM_SzNonUnif_HHH_25HUgl	
21. NGTDM_Coarse_HHH_25HUgl	
22. GLRLM_RLN_HHH_25HUgl	
23. GLRLM_RP_HHH_25HUgl	

Supplementary Table 6: For predicting recurrence at 2 years post treatment, PCA was used for feature reduction. 12 principle components, accounting for 80% of the variance, were used for modelling. Here we list the 15 radiomic features most contributing to Dimensions 1 & 2. SNS = size and shape features, GLSZM = Grey-level size zone matrix, FOS = first order statistics, GLRLM = Grey-Level Run Length Matrix, FD = fractal dimensions, NGTDM = Neighbourhood grey- tone difference matrix, GLCM = Grey-level co-occurrence matrix

Recurrence & PCA
1. GLSZM_ZonePcent_LLL_25HUgl
2. 2.GLRLM_RP_LLH_25HUgl
3. GLRLM_SRE_LHL_25HUgl
4. GLRLM_RP_LHL_25HUgl
5. FOS_ImeanAbsDev_LHH
6. FOS_Istd_HLH
7. FOS_ImeanAbsDev_HLH
8. FOS_RMS_HLH
9. GLCM_Entrop_HLH_25HUgl
10. GLCM_sumEnt_HLH_25HUgl
11. GLCM_difEnt_HLH_25HUgl
12. FOS_Istd_HHL
13. FOS_RMS_HHL
14. FOS_Istd_HHH
15. 15. FOS_RMS_HHH

Supplementary Table 7: Results of classification for the validation and external test sets. The classification threshold used was derived from the Youden Index of the validation set ROC curve for each model.

Endpoint	Model	Validation Set	External Test set	Lung 1 External Test Set
OS	**Radiomic**			
	Youden Index	0.421		
	Accuracy	0.680	0.599	0.626
	Accuracy 95% CI	0.562-0.783	0.51-0.683	0.577-0.672
	No Information Rate	0.667	0.674	0.600
	P-value (Acc > NIR)	0.457	0.973	0.148
	Sensitivity	0.680	0.651	0.787
	Specificity	0.680	0.573	0.385

	PPV	0.515	0.424	0.657
	NPV	0.810	0.773	0.546
	F1 Score	0.586	0.514	0.716
	Precision	0.515	0.424	0.657
	Recall	0.680	0.651	0.787
	**Combined**			
	Youden Index	0.399		
	Accuracy	0.627	0.568	
	Accuracy 95% CI	0.507-0.736	0.479-0.654	
	No Information Rate	0.667	0.674	
	P-value (Acc > NIR)	0.805	0.996	
	Sensitivity	0.800	0.721	
	Specificity	0.540	0.494	
	PPV	0.465	0.408	
	NPV	0.844	0.786	
	F1 Score	0.588	0.521	
	Precision	0.465	0.408	
	Recall	0.800	0.721	
**RFS**	**Radiomic**			
	Youden Index	0.509		
	Accuracy	0.773	0.705	
	Accuracy 95% CI	0.662-0.862	0.619-0.781	
	No Information Rate	0.520	0.561	
	P-value (Acc > NIR)	0.000	0.000	
	Sensitivity	0.667	0.690	
	Specificity	0.872	0.716	
	PPV	0.828	0.656	
	NPV	0.739	0.747	
	F1 Score	0.738	0.672	
	Precision	0.828	0.656	
	Recall	0.667	0.690	
	**Combined**			
	Youden Index	0.489		
	Accuracy	0.747	0.667	
	Accuracy 95% CI	0.633-0.841	0.579-0.746	
	No Information Rate	0.520	0.561	
	P-value (Acc > NIR)	0.000	0.008	
	Sensitivity	0.639	0.690	
	Specificity	0.846	0.649	
	PPV	0.793	0.606	
	NPV	0.717	0.727	
	F1 Score	0.708	0.645	
	Precision	0.793	0.606	
	Recall	0.639	0.690	
**Recurrence**	**Radiomic**			
	Youden Index	0.358		
	Accuracy	0.653	0.462	
	Accuracy 95% CI	0.535-0.76	0.375-0.551	
	No Information Rate	0.640	0.697	
	P-value (Acc > NIR)	0.457	1.000	
	Sensitivity	0.926	0.925	
	Specificity	0.500	0.261	
	PPV	0.510	0.352	
	NPV	0.923	0.889	
	F1 Score	0.658	0.510	
	Precision	0.510	0.352	
	Recall	0.926	0.925	
	**Combined**			
	Youden Index	0.337		
	Accuracy	0.613	0.508	
	Accuracy 95% CI	0.494-0.724	0.419-0.596	
	No Information Rate	0.640	0.697	
	P-value (Acc > NIR)	0.729	1.000	
	Sensitivity	0.926	0.900	
	Specificity	0.438	0.337	
	PPV	0.481	0.371	
	NPV	0.913	0.886	

	F1 Score	0.633	0.526	
	Precision	0.481	0.371	
	Recall	0.926	0.900	

Supplementary Table 8: Brier scores for the validation and external test set for each prediction model.

Endpoint	Model	Validation Set	External Test Set
Recurrence	Radiomics only	0.222	0.220
	Combined	0.224	0.197
RFS	Radiomics only	0.292	0.289
	Combined	0.295	0.291
OS	Radiomics only	0.310	0.312
	Combined	0.311	0.315

Supplementary Figure 3. Calibration curves for validation and external test sets for each prediction model. The data is divided into bins, with the *y*-axis representing the distribution of positive cases in each bin while the *x*-axis the probability as predicted by the classifier. The closer the resulting calibration curve is to the reference line. the better the model's predictions reflect the actual class distribution in the dataset.

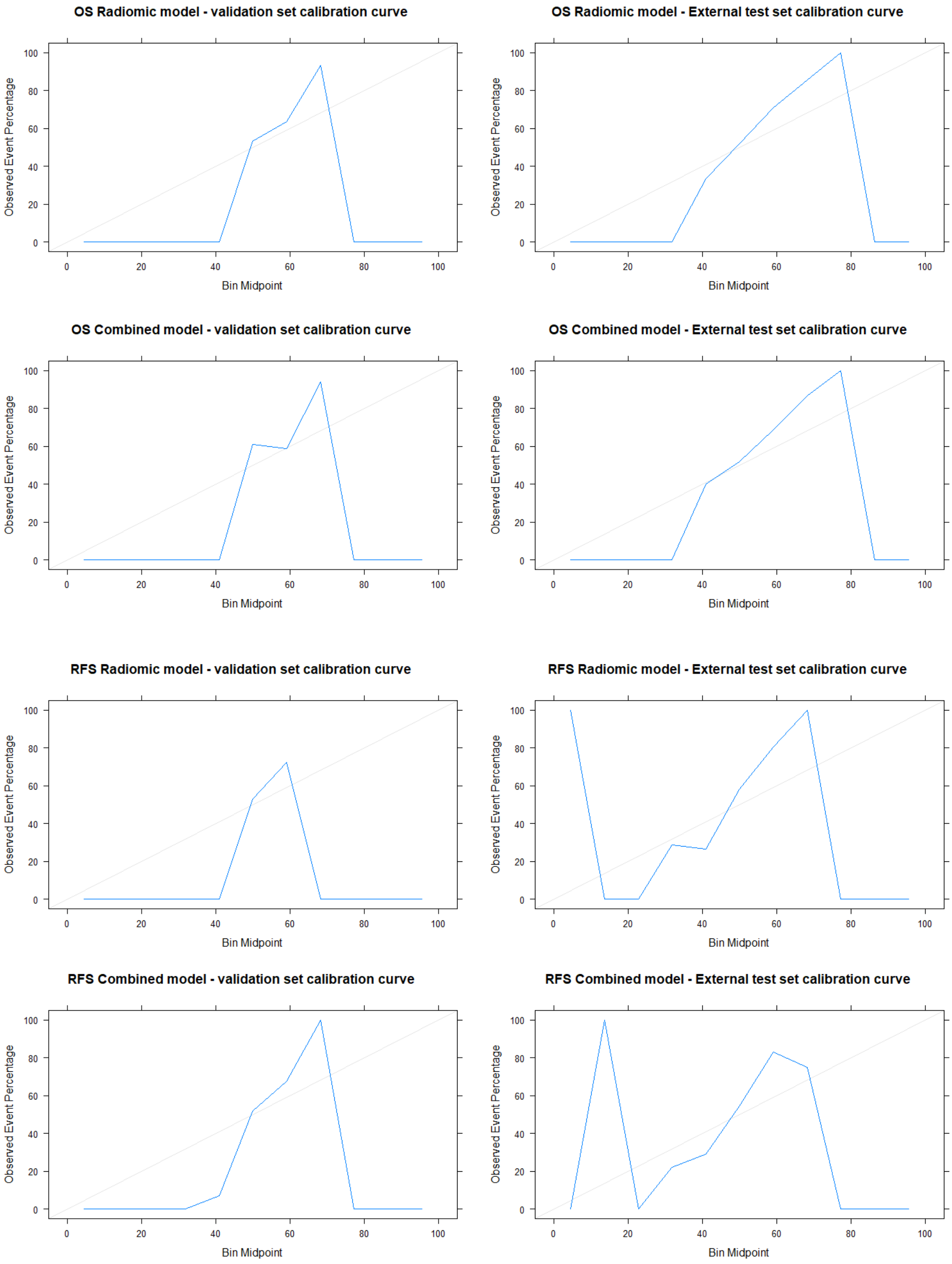

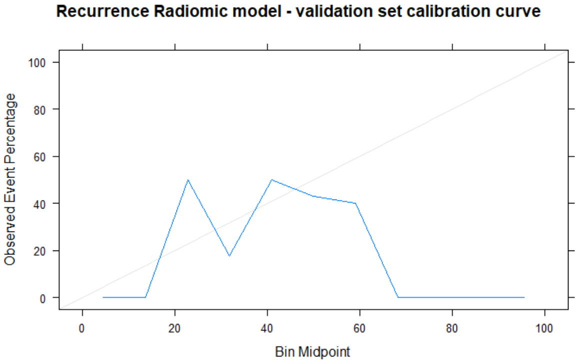
Recurrence Radiomic model - validation set calibration curve
Observed Event Percentage
Bin Midpoint
0
20
40
60
80
100

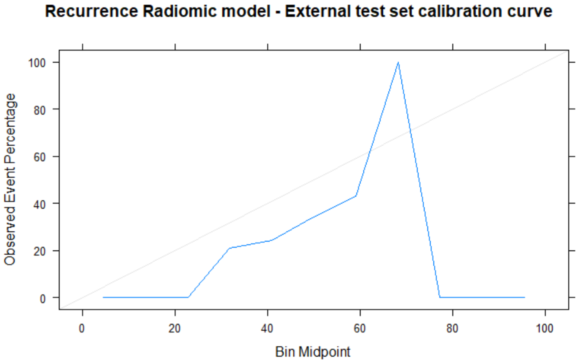
Recurrence Radiomic model - External test set calibration curve
Observed Event Percentage
Bin Midpoint
0
20
40
60
80
100

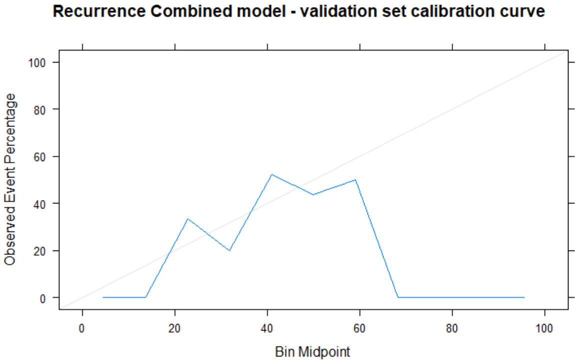
Recurrence Combined model - validation set calibration curve
Observed Event Percentage
Bin Midpoint
0
20
40
60
80
100

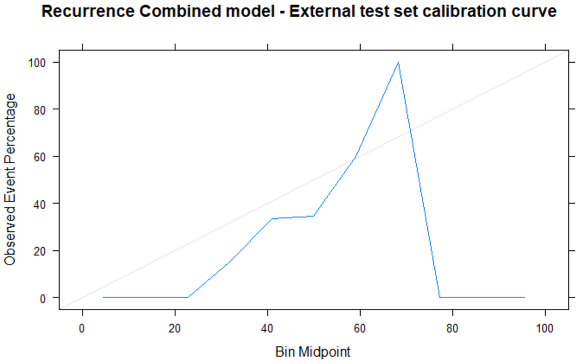
Recurrence Combined model - External test set calibration curve
Observed Event Percentage
Bin Midpoint
0
20
40
60
80
100

Supplementary Material References:

1 van Buuren S, Groothuis-Oudshoorn K. mice: Multivariate imputation by chained equations in R. *J Stat Softw* 2011; **45**: 1–67.

2 Choonghyun R. alookr: [1] Ripley, B., Venables, W. & Maintainer,]. Package 'nnet' NeedsCompilation yes. (2021).Model Classifier for Binary Classification. 2021. https://github.com/choonghyunryu/alookr/issues (accessed Aug 12, 2021).

3 Friedman J, Hastie T, Tibshirani R. Regularization paths for generalized linear models via coordinate descent. *J Stat Softw* 2010; **33**: 1–22.

4 Venables WN, Ripley BD. Generalized Linear Models. In: Modern Applied Statistics with S. New York, NY: Springer New York, 2002: 183–210.

5 Guyon I, Weston J, Barnhill S, Vapnik V. Gene selection for cancer classification using support vector machines. *Mach Learn* 2002; **46**: 389–422.

6 Battiti R. Using Mutual Information for Selecting Features in Supervised Neural Net Learning. *IEEE Trans Neural Networks* 1994; **5**: 537–50.

7 Kursa MB, Rudnicki WR. Feature selection with the boruta package. *J Stat Softw* 2010; **36**: 1–13.

8 Karatzoglou A, Hornik K, Smola A, Zeileis A. kernlab - An S4 package for kernel methods in R. *J Stat Softw* 2004; **11**: 1–20.

9 Venables WN, Ripley BD. Classification. In: Modern Applied Statistics with S. New York, NY: Springer New York, 2002: 331–51.

10 Breiman L. Random forests. *Mach Learn* 2001; **45**: 5–32.

11 Chen T, Guestrin C. XGBoost: A scalable tree boosting system. In: Proceedings of the ACM SIGKDD International Conference on Knowledge Discovery and Data Mining. New York, NY, USA: ACM, 2016: 785–94.

12 Mevik B-H, Wehrens R. Introduction to the pls Package. Help Sect. 'pls' Packag. RStudio Softw. 2015; : 1–23.

13 Riplley B, Venables B, Bates DM, Firth D, Hornik K, Gebhardt A. Package 'MASS'. Support Functions and Datasets for Venables and Ripley's MASS. Doc. Free. available internet http//www. r-project. org. 2018; : 169.

14 Michal Majka. High performance implementation of the Naive Bayes algorithm. R package naivebayes. Version 0.9.7. 2019. https://majkamichal.github.io/naivebayes/ (accessed Aug 13, 2021).

15 He H, Bai Y, Garcia EA, Li S. ADASYN: Adaptive synthetic sampling approach for imbalanced learning. In: 2008 IEEE International Joint Conference on Neural Networks (IEEE World Congress on Computational Intelligence). 2008: 1322–8.

16 Figueroa RL, Zeng-Treitler Q, Kandula S, Ngo LH. Predicting sample size required for classification performance. *BMC Med Informatics Decis Mak 2012 121* 2012; **12**: 1–10.

17 Zhang Y, Oikonomou A, Wong A, Haider MA, Khalvati F. Radiomics-based Prognosis Analysis for Non-Small Cell Lung Cancer. *Sci Rep* 2017; **7**: 1–8.

18 Lambin P, Leijenaar RTH, Deist TM, *et al.* Radiomics: The bridge between medical imaging and personalized medicine. Nat. Rev. Clin. Oncol. 2017; **14**: 749–62.

19 Park JE, Kim D, Kim HS, *et al.* Quality of science and reporting of radiomics in oncologic studies: room for improvement according to radiomics quality score and TRIPOD statement. *Eur Radiol 2019 301* 2019; **30**: 523–36.

20 Sanduleanu S, Woodruff HC, Jong EEC de, *et al.* Tracking tumor biology with radiomics: A systematic review utilizing a radiomics quality score. *Radiother Oncol* 2018; **127**: 349–60.
