# Supplementary material for: Gross tumour volume radiomics for prognostication of recurrence & death following radical radiotherapy for NSCLC
Source: NPJ Precis Oncol. 2022 Oct 27;6:77. doi: 10.1038/s41698-022-00322-3 (PMC9613990; doi:10.1038/s41698-022-00322-3)
Supplement: Supplementary file 3 — Supplemental Material [file 41698_2022_322_MOESM3_ESM.docx]

**Supplementary Material – Radiomics NSCLC Radiotherapy Prognostication & Risk-Stratification:**

**Table of Contents:**

Further details on UK datasets

Data Pre-processing for clinical data

Summary of feature reduction techniques

Summary of machine learning algorithms

Integrating clinical data & supplementary table 1

Class balancing & supplementary table 2

Training set size & supplementary figure 1

Correlation of radiomic with clinical features & supplementary figure 2

Radiomics Quality Score

TRIPOD recommendations

IBSI reporting guidelines

Supplementary Table 3 – Hyper-parameters

Supplementary Table 4 – The full list of radiomic features used in the study

Supplementary Table 5 – Radiomic features surviving feature reduction for two-year OS and RFS

Supplementary Table 6 – The 15 Radiomic features most contributing to predicting recurrence at two years post treatment

Supplementary Table 7 – Classification metrics

Supplementary Table 8 – Brier Scores

Supplementary Figure 1 – Calibration Curves

Supplementary Material References

**Further details on UK datasets:**

Each dataset was retrospectively collated from electronic patient record (EPR) and radiotherapy treatment planning systems (TPS) at UK National Health Service (NHS) Trusts:

- The RMH Dataset consists of 434 patients with stage I to III disease treated at The Royal Marsden NHS Foundation Trust with stereotactic or conventional radiotherapy with or without chemotherapy between 26/9/2014 and 23/10/2018.
- The ICHT Dataset consists of 111 patients with stage I to III disease treated at Imperial College Healthcare NHS Trust with conventional radiotherapy with or without chemotherapy between 3/2/2014 and 10/1/2019.
- The GSTT Dataset consists of 177 patients with stage I to III disease treated at Guy’s & St Thomas’ NHS Foundation Trust with stereotactic or conventional radiotherapy with or without chemotherapy between 21/1/2016 and 18/12/2018.

Eligible patients were those aged 18 or over and who received curative intent radiotherapy for NSCLC as determined by the local care team. Radiotherapy doses ranged from: 50-66Gy in 3-33 fractions encompassing a range of radical stereotactic/conventional dose-fractionation schedules.

The data were collected in early 2021, ensuring a minimum of 2 years of follow-up for all patients. Those with no known recurrence or death within 60 days of the 2 year endpoint, or no recurrence within 60 days of death, were taken to have no event. This cut-off was agreed upon by the authors after discussion about how best to reduce bias and factoring in the variable nature of clinical follow-up appointments. 60 days was taken to be mean timeframe between follow-up appointments and thus an estimated half-way point between the last time a patient was seen and the 2-year endpoint. Therefore, we agreed that if a patient had no known recurrence or death within 60 days of the endpoint, or no recurrence within 60 days of death, it was unlikely that a recurrence/death had occurred.

The following patient demographics and clinical parameters were collected: sex, age, ethnicity, World Health Organisation (WHO) performance status, smoking status, TNM8 T-stage, TNM8 N-stage, TNM8 overall clinical stage, size of primary lesion, FDG PET-CT Standard Uptake Value (SUV) of primary lesion, nodal avidity and maximal nodal SUV, whether nodes were sampled (e.g. with endoscopic bronchial ultrasound, EBUS), whether there was a confirmed pathological diagnosis (e.g. with biopsy) and histological type, body mass index, pre-treatment forced expiratory volume in 1 second (FEV1, as percent predicted) and diffusing capacity for carbon monoxide (TLCO, as percent predicted), pre and post-treatment neutrophil and lymphocyte counts, type of radiotherapy treatment received (stereotactic body radiotherapy (SBRT) or conventional radiotherapy with or without chemotherapy), total dose in Gy, number of fractions, biologically effective dose in Gy (assuming an α/β value of 10) radiotherapy gross tumour volume (GTV), radiotherapy planning target volume (PTV) and dates of radiotherapy planning scan and first and last fraction of radiotherapy. Data was pre-processed prior to machine learning as described in the Supplementary Material.

**Pre-processing for clinical data**

To meet pre-processing requirements for machine learning, categorical data were converted to numeric. One-hot-encoding converted each level of each categorical feature into a new binary feature. To mitigate for resultant increase in data dimensionality, prior to one-hot-encoding, levels of some categorical features were combined, for example, “never”, “ex” and “current” smokers were binarized to “never” and “ever”. Implicit associations between variables were made explicit: dates of planning scan and first and last fraction were replaced with the number of days between them. Missing clinical data were assumed missing at random and non-dependent on outcome. Features with more than 25% of observations missing were removed. Missing data for remaining features was imputed using the multiple imputation with chained equations (MICE) package with default arguments^1^ in R. Highly correlated features were removed using the treatment_corr function^2^, with a threshold of 0·85, removing one of each pair of correlated features (Pearson correlation for continuous and Spearman correlation for categorical features). Continuous features were standardized.

**Summary of feature reduction techniques:**

Principal Component Analysis (PCA) is an unsupervised linear transformation technique widely used for feature extraction and dimensionality reduction. PCA identifies directions of maximum variance in high-dimensional data and projects it onto a new subspace with equal or fewer dimensions. Thus, a large set of features is transformed to a smaller one that still retains most of the information in the large set.

LASSO (Least Absolute Shrinkage and Selection Operator) and Elastic Net regression are examples of regularisation methods^3^. Here a penalty is applied to the coefficient which multiplies each feature in a linear model and results in less overfitting and improved generalisation. LASSO uses the L1 regularisation penalty to force some coefficients to zero. This eliminates some features leaving a subset of predictors that are thought to be important. Alpha = 1. Elastic Net incorporates penalties from both L1 and L2 (ridge regression) regularisation. Here, Alpha =0·5. The glmnet package was used to perform both LASSO and Elastic-net regression.

Pearson, Spearman’s and Kendall’s rank correlation are “filter” feature selection methods which rely only on the characteristics of feature independently of any machine learning model. Pearson’s correlation assumes data is parametric and linear. Spearman and Kendall’s rank are non-parametric and assume a monotonic relationship between variables. Kendall’s rank is preferred to Spearman’s where dataset have a limited number of observations or contain outliers. For our study we used the corr package and specified that the top 25% (8) features be included in the feature sets following Pearson’s, Spearman’s and Kendall’s rank correlation.

Univariate LR – apply univariate logistic regression for each feature to the outcome variable and select only those features with a certain p-value. We used the glm package with a p-value <0·005, adjusted for multiple comparisons with the Benjamini & Hochberg method^4^.

RFE – Recursive Feature Elimination is a “wrapper” feature selection method which fits a model and removes the weakest feature until a specified number of features is reached. Cross-validation is used to score different feature subsets and select the best scoring collection of features to identify the optimal number of features. Features are ranked by the model’s feature importance. By recursively eliminating features iteratively the collinearity is reduced^5^. We used the rfeControl package with a random forest model and 10-fold cross validation with 5 repeats.

Mutual Information is a filter feature reduction technique that assesses relevance of a subset of features in predicting the target variable compared with redundancy with respect to other variables^6^.

Boruta is designed to act as a wrapper around a Random Forest classifier and iteratively removes features which are proved to be less relevant than random probes by statistical testing^7^.

**Summary of machine learning algorithms:**

The models used in this study are supervised classification algorithms. A summary of each is provided below.

LR – generalised linear model is a generalisation of linear regression to modelling dependencies between predictors and dependent features. Logistic regression is a form of GLM used in this study. It uses the logistic sigmoid function to return a probability value which can then be mapped to two or more separate classes^4^.

LASSO and Elastic Net regression can also be used for classification. Ridge is another form of regression where the loss function is modified to minimize the complexity of the model. Here alpha = 0.

L-SVM – linear support vector machines plot training samples and assigns a hyperplane (decision boundary) to separate these into classes. The optimal hyperplane is that which maximises the distance between data-points^8^.

KNN – K-nearest neighbours assumes that data points that are close to each other are of the same class. It takes a defined number (k) of training samples closest in Euclidian distance to a new point and predicts a class based on these^9^.

RF & XGB – Random Forest and Extreme Gradient Boosting Machines are ensemble decision-tree based models. RF uses bagging and feature variability when building each decision tree to create an uncorrelated forest whose overall prediction is more accurate than each individual tree^10^. XGB by contrast takes a boosting approach whereby trees are grown iteratively using information from a previously grown tree, to minimise the error of previous trees^11^.

PLS – Partial Least Squares is a multivariate linear regression model which forms linear combinations of features in a supervised manner. It is able to handle datasets with large numbers of features, high collinearity between features and small numbers of observations^12^.

NNET – the “nnet” package fits a single-layer feed-forward neural network^13^.

NB – the Naïve-Bayes model is based on Bayes-theorem and assumes no interdependence between variables^14^.

**Integrating clinical data**

With radiomic studies increasingly incorporating clinical data, this presents a question as to the most appropriate method for combing the different data types. We explored three approaches evaluated by validation set AUC:

1. Using the algorithm-feature reduction technique combination with the best validation set AUC as per the radiomic-only model, then concatenating clinical features to the input-matrix prior to classification
2. Using the best algorithm-feature reduction technique combination for radiomic features and the best combination for the clinical features, then ensembling these models by averaging their predictions
3. Or by ensembling these models with a Partial Least Squares (PLS) classifier.

Approach 1 resulted in the highest validation set AUC for OS (supplementary table 1) and was applied for the Combined models for RFS and recurrence also. The method resulting in the highest AUC has been utilised elsewhere as described in the main paper, however it’s important to consider that AUC alone may not be the most appropriate evaluation metric and there is a need for development of more methodology focusing on the combination of radiomic and clinical data.

| **Approach** | **1** | **2** | **3** |
| --- | --- | --- | --- |
| **OS Validation set AUC** | 0·702 | 0·6736 | 0·6784 |
| **OS Validation set AUC CI** | 0·5831-0·8217 | 0·5497-0·7975 | 0·5559-0·8009 |

**Supplementary table 1.** Exploring 3 different approaches to combine radiomic and clinical features. Results are in the form of validation set AUC and 95% CI for the OS endpoint. Approach 1 resulted in the highest validation set AUC and was selected as the approach to combine radiomic and clinical features for the Combined models for RFS and recurrence endpoints.

**Class balancing**

The OS and recurrence endpoint datasets were unbalanced (event ratios of 0.34 and 0.36, respectively). To investigate whether class balancing improved results, we applied adaptive synthetic sampling ADASYN prior to modelling and compared validation set AUCs with the original unbalanced datasets^15^. When averaging across all machine learning algorithms for Spearman (OS) and PCA (Recurrence), applying ADASYN^15^ to balance outcomes in the training set did not result in superior validation set results (supplementary table 2). The original unbalanced training sets were therefore used for modelling.

| **Endpoint** | **Recurrence** | | **OS** | |
| --- | --- | --- | --- | --- |
| **Selected feature reduction technique** | **PCA** | **PCA**  **(with ADASYN)** | **Spearman** | **Spearman**  **(with ADASYN)** |
| **XGB** | 0·623 | 0·573 | 0·667 | 0·601 |
| **RF** | 0·595 | 0·597 | 0·671 | 0·636 |
| **NB** | 0·546 | 0·511 | 0·700 | 0·700 |
| **PLS** | 0·661 | 0·649 | 0·712 | 0·716 |
| **NNET** | 0·530 | 0·545 | 0·627 | 0·604 |
| **L-SVM** | 0·532 | 0·589 | 0·550 | 0·620 |
| **LR** | 0·632 | 0·629 | 0·654 | 0·656 |
| **KNN** | 0·650 | 0·645 | 0·682 | 0·616 |
| **Ridge** | 0·634 | 0·629 | 0·708 | 0·715 |
| **LASSO** | 0·639 | 0·613 | 0·682 | 0·690 |
| **ENET** | 0·641 | 0·610 | 0·685 | 0·689 |
| **Mean** | 0·607 | 0·599 | 0·667 | 0·658 |

**Supplementary table 2.** Validation set AUCs for PCA (predicting recurrence) and Spearman (predicting OS) feature reduction techniques, with and without applying ADASYN to balance outcomes in the training set. Averaging across all machine algorithms used, ADASYN did not result in superior AUC values and therefore the original training sets were used for modelling.

**Training set size**

Training set size is an important consideration when developing radiomic-based prediction models. Data availability can often be limited in clinical studies especially when this is performed retrospectively from electronic health records. It has been suggested however that predictive power is dependent on training sample size up to a certain point, after which a classifier reaches an efficiency threshold beyond which only marginal or no improvement is seen^16^. To test robustness of our model development pipeline, we compared validation set AUCs with varying training set sizes, using the RFS endpoint with Recursive Feature Elimination and Naïve-Bayes as a random algorithm-feature reduction technique combination example. Supplementary figure 1 shows results of our experiments with varying training set size. Stability in validation set AUC is reached with training set >180 cases, thus demonstrating that our n=302 appears sufficient. At approximately 125 cases AUC drops by ~3%, a change which could be observed by chance as different cases enter the training set at random. As training set size increases, the overall trend of increasing AUC emerges. This is also seen in^17^.


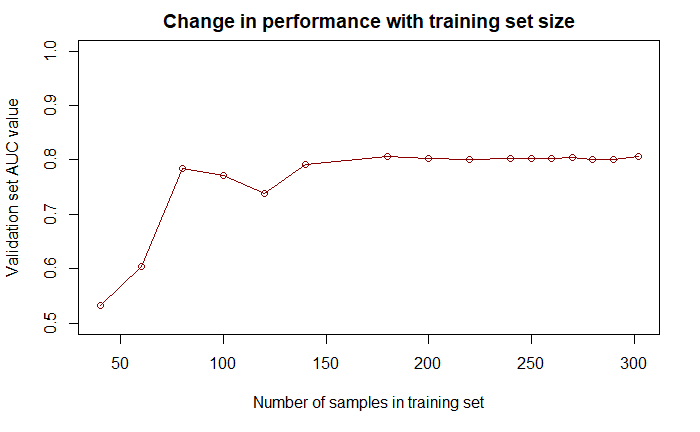


**Supplementary figure 1.** Validation set AUC values with varying training set size. Values stabilise once training set size reaches beyond 180 cases.

**Correlation of Radiomic with Clinical Features**

We explored the association between the radiomic features used in each model (OS, RFS, recurrence, as detailed in Supplementary Tables 5 & 6 below), with the clinical features. Correlation heatmaps are shown in Supplementary Figure 2. For OS, there was a strong association between T1-stage and NGLDM_Coarseness, a texture feature that measures the level of spatial rate of change in intensity. Size of primary tumour (which correlates with T-stage) was also strongly associated with other texture features including Grey-level run-length matrix (GLRLM), Gray Level Size Zone (GLSZM) and Gray Level Co-occurrence Matrix (GLCM) features. Interestingly, a weak association between GLSZM is also noted with nodal avidity and nodal SUV, though this may be due to larger tumours more likely having advanced nodal involvement. For predicting RFS, size of primary tumour was strongly associated with the same texture features as for OS, as well as the median gray level intensity within the ROI (FOS_lmedian_LLL). T1-stage disease was associated with the square-root of the mean of all the squared intensity values (FOS_RMS). With recurrence, the strongest associations were between T1-stage and GLSZM Zone Percentage, which measures the coarseness of the texture by taking the ratio of number of zones and number of voxels in the ROI, and GLRLM..


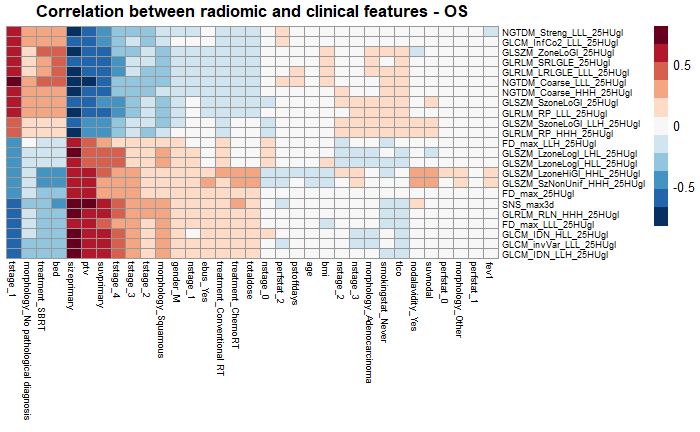

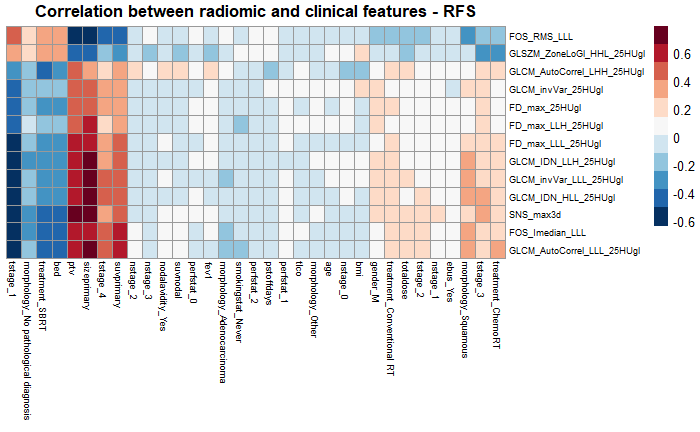

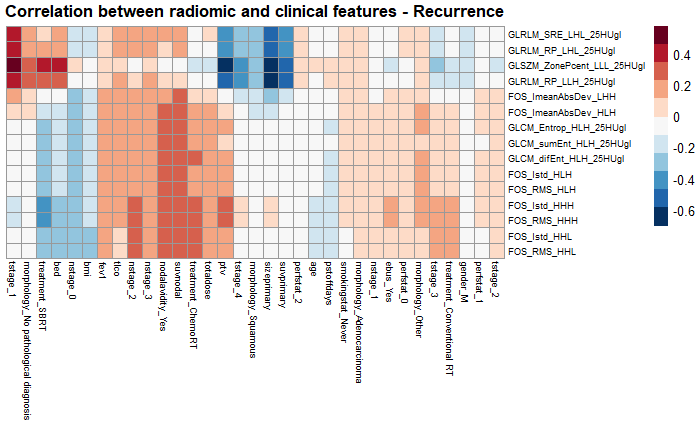


Supplementary Figure 2. Correlation heatmaps of the radiomic features used for modelling each endpoint, against clinical features. Positive associations are in red and negative in blue.

**The Radiomics Quality Score**^18^ for our study is tabulated below. Our study scores comparatively high according to two studies evaluating studies against the radiomics quality score^19,20^.

| **Criteria** | **Points** | **Our Score** |
| --- | --- | --- |
| 1. Image protocol quality – well-documented image protocols (for example, contrast, slice thickness, energy, etc.) and/or usage of public image protocols allow reproducibility/replicability | + 1 (if protocols are well-documented) + 1 (if public protocol is used) | 0 |
| 1. Multiple segmentations – possible actions are: segmentation by different physicians/algorithms/software, perturbing segmentations by (random) noise, segmentation at different breathing cycles. Analyse feature robustness to segmentation variabilities | + 1 | 0 |
| 1. Phantom study on all scanners – detect inter-scanner differences and vendor-dependent features. Analyse feature robustness to these sources of variability | + 1 | 0 |
| 1. Imaging at multiple time points – collect images of individuals at additional time points. Analyse feature robustness to temporal variabilities (for example, organ movement, organ expansion/shrinkage) | + 1 | 0 |
| 1. Feature reduction or adjustment for multiple testing – decreases the risk of overfitting. Overfitting is inevitable if the number of features exceeds the number of samples. Consider feature robustness when selecting features | − 3 (if neither measure is implemented) + 3 (if either measure is implemented) | 3 |
| 1. Multivariable analysis with non-radiomics features (for example, EGFR mutation) – is expected to provide a more holistic model. Permits correlating/inferencing between radiomics and non-radiomics features | + 1 | 1 |
| 1. Detect and discuss biological correlates – demonstration of phenotypic differences (possibly associated with underlying gene–protein expression patterns) deepens understanding of radiomics and biology | + 1 | 1 |
| 1. Cut-off analyses – determine risk groups by either the median, a previously published cut-off or report a continuous risk variable. Reduces the risk of reporting overly optimistic results | + 1 | 1 |
| 1. Discrimination statistics – report discrimination statistics (for example, C-statistic, ROC curve, AUC) and their statistical significance (for example, p-values, confidence intervals). One can also apply resampling method (for example, bootstrapping, cross-validation) | + 1 (if a discrimination statistic and its statistical significance are reported) + 1 (if a resampling method technique is also applied) | 2 |
| 1. Calibration statistics – report calibration statistics (for example, Calibration-in-the-large/slope, calibration plots) and their statistical significance (for example, *P*-values, confidence intervals). One can also apply resampling method (for example, bootstrapping, cross-validation) | + 1 (if a calibration statistic and its statistical significance are reported) + 1 (if a resampling method technique is also applied) | 2 |
| 1. Prospective study registered in a trial database – provides the highest level of evidence supporting the clinical validity and usefulness of the radiomics biomarker | + 7 (for prospective validation of a radiomics signature in an appropriate trial) | 0 |
| 1. Validation – the validation is performed without retraining and without adaptation of the cut-off value, provides crucial information with regard to credible clinical performance | - 5 (if validation is missing) + 2 (if validation is based on a dataset from the same institute) + 3 (if validation is based on a dataset from another institute) + 4 (if validation is based on two datasets from two distinct institutes) + 4 (if the study validates a previously published signature) + 5 (if validation is based on three or more datasets from distinct institutes) *Datasets should be of comparable size and should have at least 10 events per model feature | 4 |
| 1. Comparison to ‘gold standard’ – assess the extent to which the model agrees with/is superior to the current ‘gold standard’ method (for example, TNM-staging for survival prediction). This comparison shows the added value of radiomics | + 2 | 2 |
| 1. Potential clinical utility – report on the current and potential application of the model in a clinical setting (for example, decision curve analysis). | + 2 | 2 |
| 1. Cost-effectiveness analysis - report on the cost-effectiveness of the clinical application (for example, QALYs generated) | + 1 | 0 |
| 1. Open science and data - make code and data publicly available. Open science facilitates knowledge transfer and reproducibility of the study | + 1 (if scans are open source) + 1 (if region of interest segmentations are open source) + 1 (if code is open source) + 1 (if radiomics features are calculated on a set of representative ROIs and the calculated features and representative ROIs are open source) | 0 |
| Total points (36 = 100%) | 36 | 18 (50%) |

**TRIPOD recommendations.** An “X” indicates if the objective is addressed in the study:

| **Section/Topic** | **Item** | **Checklist Item** | **Page** |
| --- | --- | --- | --- |
| **Title and abstract CT Radiomics Models for Prognostication and Risk-Stratification of Recurrence & Death after Curative-Intent Radiotherapy for Non-Small Cell Lung Cancer** | | | |
| Title | 1 | Identify the study as developing and/or validating a multivariable prediction model, the target population, and the outcome to be predicted. | 1 |
| Abstract | 2 | Provide a summary of objectives, study design, setting, participants, sample size, predictors, outcome, statistical analysis, results, and conclusions. | 1 |
| **Introduction** | | | |
| Background and objectives | 3a | Explain the medical context (including whether diagnostic or prognostic) and rationale for developing or validating the multivariable prediction model, including references to existing models. | 3 |
|  | 3b | Specify the objectives, including whether the study describes the development or validation of the model or both. | 3 |
| **Methods** | | | |
| Source of data | 4a | Describe the study design or source of data (e.g., randomized trial, cohort, or registry data), separately for the development and validation data sets, if applicable. | 4 |
|  | 4b | Specify the key study dates, including start of accrual; end of accrual; and, if applicable, end of follow-up. | 4/SM |
| Participants | 5a | Specify key elements of the study setting (e.g., primary care, secondary care, general population) including number and location of centres. | 4/SM |
|  | 5b | Describe eligibility criteria for participants. | 4/SM |
|  | 5c | Give details of treatments received, if relevant. | 4/SM |
| Outcome | 6a | Clearly define the outcome that is predicted by the prediction model, including how and when assessed. | 4 |
|  | 6b | Report any actions to blind assessment of the outcome to be predicted. |  |
| Predictors | 7a | Clearly define all predictors used in developing or validating the multivariable prediction model, including how and when they were measured. | SM |
|  | 7b | Report any actions to blind assessment of predictors for the outcome and other predictors. |  |
| Sample size | 8 | Explain how the study size was arrived at. |  |
| Missing data | 9 | Describe how missing data were handled (e.g., complete-case analysis, single imputation, multiple imputation) with details of any imputation method. | SM |
| Statistical analysis methods | 10a | Describe how predictors were handled in the analyses. | 4 |
|  | 10b | Specify type of model, all model-building procedures (including any predictor selection), and method for internal validation. | 4 |
|  | 10d | Specify all measures used to assess model performance and, if relevant, to compare multiple models. | 5 |
| Risk groups | 11 | Provide details on how risk groups were created, if done. | 6 |
| **Results** | | | |
| Participants | 13a | Describe the flow of participants through the study, including the number of participants with and without the outcome and, if applicable, a summary of the follow-up time. A diagram may be helpful. | 4 |
|  | 13b | Describe the characteristics of the participants (basic demographics, clinical features, available predictors), including the number of participants with missing data for predictors and outcome. | 6 |
| Model development | 14a | Specify the number of participants and outcome events in each analysis. | 6 |
|  | 14b | If done, report the unadjusted association between each candidate predictor and outcome. |  |
| Model specification | 15a | Present the full prediction model to allow predictions for individuals (i.e., all regression coefficients, and model intercept or baseline survival at a given time point). |  |
|  | 15b | Explain how to the use the prediction model. |  |
| Model performance | 16 | Report performance measures (with CIs) for the prediction model. | 8 |
| **Discussion** | | | |
| Limitations | 18 | Discuss any limitations of the study (such as nonrepresentative sample, few events per predictor, missing data). | 16 |
| Interpretation | 19b | Give an overall interpretation of the results, considering objectives, limitations, and results from similar studies, and other relevant evidence. | 15 |
| Implications | 20 | Discuss the potential clinical use of the model and implications for future research. | 16 |
| **Other information** | | | |
| Supplementary information | 21 | Provide information about the availability of supplementary resources, such as study protocol, Web calculator, and data sets. | 17/SM |
| Funding | 22 | Give the source of funding and the role of the funders for the present study. | 6 |
| SM = Supplementary Material | | | |

**IBSI reporting guidelines:** “Y” indicates which sections are reported in the manuscript. Sections not relating to CT imaging are marked as not applicable (“NA”).

| IBSI Reporting Guidelines | | | | |
| --- | --- | --- | --- | --- |
| Patient | | | | |
| **Topic** | **Modality** | **Item** | **Description** | **Included in manuscript** |
| Region of interest [[1]](https://ibsi.readthedocs.io/en/latest/04_Radiomics_reporting_guidelines_and_nomenclature.html#id17) |  | 1 | Describe the region of interest that is being imaged. | Y |
| Patient preparation |  | 2a | Describe specific instructions given to patients prior to image acquisition, e.g. fasting prior to imaging. |  |
|  |  | 2b | Describe administration of drugs to the patient prior to image acquisition, e.g. muscle relaxants. |  |
|  |  | 2c | Describe the use of specific equipment for patient comfort during scanning, e.g. ear plugs. |  |
| Radioactive tracer | PET, SPECT | 3a | Describe which radioactive tracer was administered to the patient, e.g. 18F-FDG. | NA |
|  | PET, SPECT | 3b | Describe the administration method. | NA |
|  | PET, SPECT | 3c | Describe the injected activity of the radioactive tracer at administration. | NA |
|  | PET, SPECT | 3d | Describe the uptake time prior to image acquisition. | NA |
|  | PET, SPECT | 3e | Describe how competing substance levels were controlled. [[2]](https://ibsi.readthedocs.io/en/latest/04_Radiomics_reporting_guidelines_and_nomenclature.html#id18) | NA |
| Contrast agent |  | 4a | Describe which contrast agent was administered to the patient. |  |
|  |  | 4b | Describe the administration method. |  |
|  |  | 4c | Describe the injected quantity of contrast agent. |  |
|  |  | 4d | Describe the uptake time prior to image acquisition. |  |
|  |  | 4e | Describe how competing substance levels were controlled. |  |
| Comorbidities |  | 5 | Describe if the patients have comorbidities that affect imaging. [[3]](https://ibsi.readthedocs.io/en/latest/04_Radiomics_reporting_guidelines_and_nomenclature.html#id19) |  |
| Acquisition | | | | |
| **Topic** | **Modality** | **Item** | **Description** | **Included** |
| Acquisition protocol |  | 6 | Describe whether a standard imaging protocol was used, and where its description may be found. |  |
| Scanner type |  | 7 | Describe the scanner type(s) and vendor(s) used in the study. |  |
| Imaging modality |  | 8 | Clearly state the imaging modality that was used in the study, e.g. CT, MRI. |  |
| Static/dynamic scans |  | 9a | State if the scans were static or dynamic. |  |
|  | Dynamic scans | 9b | Describe the acquisition time per time frame. |  |
|  | Dynamic scans | 9c | Describe any temporal modelling technique that was used. |  |
| Scanner calibration |  | 10 | Describe how and when the scanner was calibrated. |  |
| Patient instructions |  | 11 | Describe specific instructions given to the patient during acquisition, e.g. breath holding. |  |
| Anatomical motion correction |  | 12 | Describe the method used to minimise the effect of anatomical motion. |  |
| Scan duration |  | 13 | Describe the duration of the complete scan or the time per bed position. |  |
| Tube voltage | CT | 14 | Describe the peak kilo voltage output of the X-ray source. |  |
| Tube current | CT | 15 | Describe the tube current in mA. |  |
| Time-of-flight | PET | 16 | State if scanner time-of-flight capabilities are used during acquisition. | NA |
| RF coil | MRI | 17 | Describe what kind RF coil used for acquisition, incl. vendor. | NA |
| Scanning sequence | MRI | 18a | Describe which scanning sequence was acquired. | NA |
|  | MRI | 18b | Describe which sequence variant was acquired. | NA |
|  | MRI | 18c | Describe which scan options apply to the current sequence, e.g. flow compensation, cardiac gating. | NA |
| Repetition time | MRI | 19 | Describe the time in ms between subsequent pulse sequences. | NA |
| Echo time | MRI | 20 | Describe the echo time in ms. | NA |
| Echo train length | MRI | 21 | Describe the number of lines in k-space that are acquired per excitation pulse. | NA |
| Inversion time | MRI | 22 | Describe the time in ms between the middle of the inverting RF pulse to the middle of the excitation pulse. | NA |
| Flip angle | MRI | 23 | Describe the flip angle produced by the RF pulses. | NA |
| Acquisition type | MRI | 24 | Describe the acquisition type of the MRI scan, e.g. 3D. | NA |
| k-space traversal | MRI | 25 | Describe the acquisition trajectory of the k-space. | NA |
| Number of averages/ excitations | MRI | 26 | Describe the number of times each point in k-space is sampled. | NA |
| Magnetic field strength | MRI | 27 | Describe the nominal strength of the MR magnetic field. | NA |
| Reconstruction | | | | |
| **Topic** | **Modality** | **Item** | **Description** | **Included** |
| In-plane resolution |  | 28 | Describe the distance between pixels, or alternatively the field of view and matrix size. |  |
| Image slice thickness |  | 29 | Describe the slice thickness. | Y |
| Image slice spacing |  | 30 | Describe the distance between image slices. [[6]](https://ibsi.readthedocs.io/en/latest/04_Radiomics_reporting_guidelines_and_nomenclature.html#id22) |  |
| Convolution kernel | CT | 31a | Describe the convolution kernel used to reconstruct the image. |  |
|  | CT | 31b | Describe settings pertaining to iterative reconstruction algorithms. |  |
| Exposure | CT | 31c | Describe the exposure (in mAs) in slices containing the region of interest. |  |
| Reconstruction method | PET | 32a | Describe which reconstruction method was used, e.g. 3D OSEM. | NA |
|  | PET | 32b | Describe the number of iterations for iterative reconstruction. | NA |
|  | PET | 32c | Describe the number of subsets for iterative reconstruction. | NA |
| Point spread function modelling | PET | 33 | Describe if and how point-spread function modelling was performed. | NA |
| Image corrections | PET | 34a | Describe if and how attenuation correction was performed. | NA |
|  | PET | 34b | Describe if and how other forms of correction were performed, e.g. scatter correction, randoms correction, dead time correction etc. | NA |
| Reconstruction method | MRI | 35a | Describe the reconstruction method used to reconstruct the image from the k-space information. | NA |
|  | MRI | 35b | Describe any artifact suppression methods used during reconstruction to suppress artifacts due to undersampling of k-space. | NA |
| Diffusion-weigh ted imaging | DWI-MRI | 36 | Describe the b-values used for diffusion-weigh ting. | NA |
| Image registration | | | | |
| **Topic** | **Modality** | **Item** | **Description** | **Included** |
| Registration method |  | 37 | Describe the method used to register multi-modality imaging. | NA |
| Image processing | | | | |
| Data conversion | | | | |
| **Topic** | **Modality** | **Item** | **Description** | **Included** |
| SUV normalisation | PET | 38 | Describe which standardised uptake value (SUV) normalisation method is used. | NA |
| ADC computation | DWI-MRI | 39 | Describe how apparent diffusion coefficient (ADC) values were calculated. | NA |
| Other data conversions |  | 40 | Describe any other conversions that are performed to generate e.g. perfusion maps. | NA |
| Post-acquisition processsing | | | | |
| **Topic** | **Modality** | **Item** | **Description** | **Included** |
| Anti-aliasing |  | 41 | Describe the method used to deal with anti-aliasing when down-sampling during interpolation. | Y |
| Noise suppression |  | 42 | Describe methods used to suppress image noise. |  |
| Post-reconstruc tion smoothing filter | PET | 43 | Describe the width of the Gaussian filter (FWHM) to spatially smooth intensities. | NA |
| Skull stripping | MRI (brain) | 44 | Describe method used to perform skull stripping. | NA |
| Non-uniformity correction [[7]](https://ibsi.readthedocs.io/en/latest/04_Radiomics_reporting_guidelines_and_nomenclature.html#id23) | MRI | 45 | Describe the method and settings used to perform non-uniformity correction. | NA |
| Intensity normalisation |  | 46 | Describe the method and settings used to normalise intensity distributions within a patient or patient cohort. |  |
| Other post-acquisitio n processing methods |  | 47 | Describe any other methods that were used to process the image and are not mentioned separately in this list. | Y |
| Segmentation | | | | |
| **Topic** | **Modality** | **Item** | **Description** | **Included** |
| Segmentation method |  | 48a | Describe how regions of interest were segmented, e.g. manually. | Y |
|  |  | 48b | Describe the number of experts, their expertise and consensus strategies for manual delineation. | Y |
|  |  | 48c | Describe methods and settings used for semi-automatic and fully automatic segmentation. | NA |
|  |  | 48d | Describe which image was used to define segmentation in case of multi-modality imaging. | NA |
| Conversion to mask |  | 49 | Describe the method used to convert polygonal or mesh-based segmentations to a voxel-based mask. | NA |
| Image interpolation | | | | |
| **Topic** | **Modality** | **Item** | **Description** | **Included** |
| Interpolation method |  | 50a | Describe which interpolation algorithm was used to interpolate the image. | Y |
|  |  | 50b | Describe how the position of the interpolation grid was defined, e.g. align by center. | Y |
|  |  | 50c | Describe how the dimensions of the interpolation grid were defined, e.g. rounded to nearest integer. | Y |
|  |  | 50d | Describe how extrapolation beyond the original image was handled. | NA |
| Voxel dimensions |  | 51 | Describe the size of the interpolated voxels. | Y |
| Intensity rounding | CT | 52 | Describe how fractional Hounsfield Units are rounded to integer values after interpolation. | Y |
| ROI interpolation | | | | |
| **Topic** | **Modality** | **Item** | **Description** | **Included** |
| Interpolation method |  | 53 | Describe which interpolation algorithm was used to interpolate the region of interest mask. | Y |
| Partially masked voxels |  | 54 | Describe how partially masked voxels after interpolation are handled. | Y |
| Re-segmentation | | | | |
| **Topic** | **Modality** | **Item** | **Description** | **Included** |
| Re-segmentation methods |  | 55 | Describe which methods and settings are used to re-segment the ROI intensity mask. | NA |
| Discretisation | | | | |
| **Topic** | **Modality** | **Item** | **Description** | **Included** |
| Discretisation method [[8]](https://ibsi.readthedocs.io/en/latest/04_Radiomics_reporting_guidelines_and_nomenclature.html#id24) |  | 56a | Describe the method used to discretise image intensities. | Y |
|  |  | 56b | Describe the number of bins (FBN) or the bin size (FBS) used for discretisation. | Y |
|  |  | 56c | Describe the lowest intensity in the first bin for FBS discretisation. |  |
| Image transformation | | | | |
| **Topic** | **Modality** | **Item** | **Description** | **Included** |
| Image filter [[10]](https://ibsi.readthedocs.io/en/latest/04_Radiomics_reporting_guidelines_and_nomenclature.html#id26) |  | 57 | Describe the methods and settings used to filter images, e.g. Laplacian-of-Ga ussian. | Y |
| Image biomarker computation | | | | |
| **Topic** | **Modality** | **Item** | **Description** | **Included** |
| Biomarker set |  | 58 | Describe which set of image biomarkers is computed and refer to their definitions or provide these. | Y |
| IBSI compliance |  | 59 | State if the software used to extract the set of image biomarkers is compliant with the IBSI benchmarks. | Y |
| Robustness |  | 60 | Describe how robustness of the image biomarkers was assessed, e.g. test-retest analysis. | Y |
| Software availability |  | 61 | Describe which software and version was used to compute image biomarkers. | Y |
| Image biomarker computation - texture parameters | | | | |
| **Topic** | **Modality** | **Item** | **Description** | **Included** |
| Texture matrix aggregation |  | 62 | Define how texture-matrix based biomarkers were computed from underlying texture matrices. | Y |
| Distance weighting |  | 63 | Define how CM, RLM, NGTDM and NGLDM weight distances, e.g. no weighting. |  |
| CM symmetry |  | 64 | Define whether symmetric or asymmetric co-occurrence matrices were computed. |  |
| CM distance |  | 65 | Define the (Chebyshev) distance at which co-occurrence of intensities is determined, e.g. 1. |  |
| SZM linkage distance |  | 66 | Define the distance and distance norm for which voxels with the same intensity are considered to belong to the same zone for the purpose of constructing an SZM, e.g. Chebyshev distance of 1. |  |
| DZM linkage distance |  | 67 | Define the distance and distance norm for which voxels with the same intensity are considered to belong to the same zone for the purpose of constructing a DZM, e.g. Chebyshev distance of 1. |  |
| DZM zone distance norm |  | 68 | Define the distance norm for determining the distance of zones to the border of the ROI, e.g. Manhattan distance. |  |
| NGTDM distance |  | 69 | Define the neighbourhood distance and distance norm for the NGTDM, e.g. Chebyshev distance of 1. |  |
| NGLDM distance |  | 70 | Define the neighbourhood distance and distance norm for the NGLDM, e.g. Chebyshev distance of 1. |  |
| NGLDM coarseness |  | 71 | Define the coarseness parameter for the NGLDM, e.g. 0. |  |
| Machine learning and radiomics analysis | | | | |
| **Topic** | **Modality** | **Item** | **Description** | **Included** |
| Diagnostic and prognostic modelling |  | 72 | See the TRIPOD guidelines for reporting on diagnostic and prognostic modelling. | Y |
| Comparison with known factors |  | 73 | Describe where performance of radiomics models is compared with known (clinical) factors. | Y |
| Multicollineari ty |  | 74 | Describe where the multicollineari ty between image biomarkers in the signature is assessed. | Y |
| Model availability |  | 75 | Describe where radiomics models with the necessary pre-processing information may be found. | Y |
| Data availability |  | 76 | Describe where imaging data and relevant meta-data used in the study may be found. | Y |

**Supplementary Table 3:** Hyperparameters used for the Radiomic and Combined models. Algorithms used were PLS (predicting OS and RFS) and KNN, PLS and E-Net for the ensemble model (predicting recurrence).

| **Endpoint** | **Model** | **Algorithm** | **Library** | **Hyperparameter** |
| --- | --- | --- | --- | --- |
| OS | Radiomic | PLS | *pls* | ncomp = 1 |
|  | Combined | PLS | *pls* | ncomp = 1 |
| RFS | Radiomic | PLS | *pls* | ncomp = 1 |
|  | Combined | PLS | *pls* | ncomp = 1 |
| Recurrence | Radiomic | KNN | *knn* | k = 27 |
|  |  | PLS | *pls* | ncomp = 1 |
|  |  | E-Net | *glmnet* | alpha = 0·5, lambda = 0·0199951 |
|  | Combined | KNN | *knn* | k = 29 |
|  |  | PLS | *pls* | ncomp = 1 |
|  |  | E-Net | *glmnet* | alpha = 0·5, lambda = 0·03029972 |

**Supplementary Table 4:** The full list of radiomic features used in the study.

| Technique (Total number) | Features |
| --- | --- |
| First order statistics (FOS)  (15) | 1. Coefficient of Variation 2. Mean 3. Median 4. Mode 5. Standard Deviation 6. Minimum 7. Maximum 8. Range 9. Skewness 10. Kurtosis 11. Mean Absolute Deviation 12. Root mean square 13. Area under the Curve 14. Entropy 15. Energy |
| Grey-level co-occurrence matrix (GLCM)  (23) | 1. Variance 2. Correlation 3. Information Measure of Correlation 1) 4. Information Measure of Correlation 2) 5. Cluster Shade 6. Cluster Prominence 7. Angular Second Moment 8. Maximum Probability 9. Entropy 10. Contrast 11. Dissimilarity 12. Homogeneity 13. Sum Average 14. Sum Variance 15. Sum Entropy 16. Difference in Variance 17. Difference entropy 18. Autocorrelation 19. Cluster Tendency 20. Homogeneity 1 21. Inverse Difference Moment Normalised 22. Inverse Difference Normalised 23. Inverse Variance |
| Grey-level size zone matrix (GLSZM)  (13) | 1. Small Zone Emphasis 2. Large Zone Emphasis 3. Grey-level Non Uniformity 4. Size zone Non uniformity 5. Zone Percentage 6. Zone Low grey-level Emphasis 7. Zone Low grey-level Emphasis 8. Small Zone Low grey level Zone Emphasis 9. Small Zone High grey level Zone Emphasis 10. Large Zone Low grey level Emphasis 11. Large Zone High grey level Emphasis 12. Grey Level Variance 13. Size-Zone Variance |
| Neighborhood grey- tone difference matrix (NGTDM)  (5) | 1. Coarseness 2. Contrast 3. Busyness 4. Complex 5. Strength |
| Size and Shape (SNS)  (8) | 1. Volume 2. Area 3. Surface to volume ratio 4. Sphericity 5. Spherical disproportion 6. Compactness 1 7. Compactness 2 8. Maximum 3d diameter |
| Grey-Level Run Length Matrix (GLRLM)  (11) | 1. Short Run Emphasis 2. Long Run Emphasis 3. Grey-Level Non-Uniformity 4. Run Length Non-Uniformity 5. Run Percentage 6. Low Grey-Level Run Emphasis 7. High Grey Level Run Emphasis 8. Short Run Low Grey Level Emphasis 9. Short Run High Grey Level Emphasis 10. Long Run Low Grey Level Emphasis 11. Long Run High Grey Level Emphasis |
| Fractal Dimension (FD)  (6) | 1. Mean 2. Standard deviation 3. Variance 4. Lacunarity 5. Maximum 6. Minimum |
| Wavelet transformations x8 | As above but with 8 filters |

**Supplementary Table 5:** The list of radiomic features surviving feature reduction for OS and RFS. Feature reduction techniques used are Spearman and Pearson’s rank correlation, selecting the top 20% of features remaining after first removing highly correlated features and applying a univariate logistic regression. SNS = size and shape features, GLSZM = Grey-level size zone matrix, FOS = first order statistics, GLRLM = Grey-Level Run Length Matrix, FD = fractal dimensions, NGTDM = Neighbourhood grey- tone difference matrix, GLCM = Grey-level co-occurrence matrix

| **OS & Spearman’s rank correlation** | **RFS & Pearson’s rank correlation** |
| --- | --- |
| 1. SNS_max3d 2. GLSZM_ZoneLoGl_25HUgl 3. GLSZM_SzoneLoGl_25HUgl 4. GLRLM_SRLGLE_25HUgl 5. FD_max_25HUgl 6. NGTDM_Coarse_LLL_25HUgl 7. NGTDM_Streng_LLL_25HUgl 8. GLRLM_RP_LLL_25HUgl 9. GLRLM_LRLGLE_LLL_25HUgl 10. FD_max_LLL_25HUgl 11. GLCM_InfCo2_LLL_25HUgl 12. GLCM_invVar_LLL_25HUgl 13. GLSZM_SzoneLoGl_LLH_25HUgl 14. FD_max_LLH_25HUgl 15. GLCM_IDN_LLH_25HUgl 16. GLSZM_LzoneLogl_LHL_25HUgl 17. GLSZM_LzoneLogl_HLL_25HUgl 18. GLCM_IDN_HLL_25HUgl 19. GLSZM_LzoneHiGl_HHL_25HUgl 20. GLSZM_SzNonUnif_HHH_25HUgl 21. NGTDM_Coarse_HHH_25HUgl 22. GLRLM_RLN_HHH_25HUgl 23. GLRLM_RP_HHH_25HUgl | 1. SNS_max3d 2. FD_max_25HUgl 3. GLCM_invVar_25HUgl 4. FOS_Imedian_LLL 5. FOS_RMS_LLL 6. FD_max_LLL_25HUgl 7. GLCM_AutoCorrel_LLL_25HUgl 8. GLCM_invVar_LLL_25HUgl 9. FD_max_LLH_25HUgl 10. GLCM_IDN_LLH_25HUgl 11. GLCM_AutoCorrel_LHH_25HUgl 12. GLCM_IDN_HLL_25HUgl 13. GLSZM_ZoneLoGl_HHL_25HUgl |

**Supplementary Table 6:** For predicting recurrence at 2 years post treatment, PCA was used for feature reduction. 12 principle components, accounting for 80% of the variance, were used for modelling. Here we list the 15 radiomic features most contributing to Dimensions 1 & 2. SNS = size and shape features, GLSZM = Grey-level size zone matrix, FOS = first order statistics, GLRLM = Grey-Level Run Length Matrix, FD = fractal dimensions, NGTDM = Neighbourhood grey- tone difference matrix, GLCM = Grey-level co-occurrence matrix

| **Recurrence & PCA** |
| --- |
| 1. GLSZM_ZonePcent_LLL_25HUgl 2. 2.GLRLM_RP_LLH_25HUgl 3. GLRLM_SRE_LHL_25HUgl 4. GLRLM_RP_LHL_25HUgl 5. FOS_ImeanAbsDev_LHH 6. FOS_Istd_HLH 7. FOS_ImeanAbsDev_HLH 8. FOS_RMS_HLH 9. GLCM_Entrop_HLH_25HUgl 10. GLCM_sumEnt_HLH_25HUgl 11. GLCM_difEnt_HLH_25HUgl 12. FOS_Istd_HHL 13. FOS_RMS_HHL 14. FOS_Istd_HHH 15. 15. FOS_RMS_HHH |

**Supplementary Table 7:** Results of classification for the validation and external test sets. The classification threshold used was derived from the Youden Index of the validation set ROC curve for each model.

| **Endpoint** | **Model** | **Validation Set** | **External Test set** | **Lung 1 External Test Set** |
| --- | --- | --- | --- | --- |
| **OS** | **Radiomic** |  |  |  |
|  | Youden Index | 0.421 |  |  |
|  | Accuracy | 0.680 | 0.599 | 0.626 |
|  | Accuracy 95% CI | 0.562-0.783 | 0.51-0.683 | 0.577-0.672 |
|  | No Information Rate | 0.667 | 0.674 | 0.600 |
|  | P-value (Acc > NIR) | 0.457 | 0.973 | 0.148 |
|  | Sensitivity | 0.680 | 0.651 | 0.787 |
|  | Specificity | 0.680 | 0.573 | 0.385 |
|  | PPV | 0.515 | 0.424 | 0.657 |
|  | NPV | 0.810 | 0.773 | 0.546 |
|  | F1 Score | 0.586 | 0.514 | 0.716 |
|  | Precision | 0.515 | 0.424 | 0.657 |
|  | Recall | 0.680 | 0.651 | 0.787 |
|  | **Combined** |  |  |  |
|  | Youden Index | 0.399 |  |  |
|  | Accuracy | 0.627 | 0.568 |  |
|  | Accuracy 95% CI | 0.507-0.736 | 0.479-0.654 |  |
|  | No Information Rate | 0.667 | 0.674 |  |
|  | P-value (Acc > NIR) | 0.805 | 0.996 |  |
|  | Sensitivity | 0.800 | 0.721 |  |
|  | Specificity | 0.540 | 0.494 |  |
|  | PPV | 0.465 | 0.408 |  |
|  | NPV | 0.844 | 0.786 |  |
|  | F1 Score | 0.588 | 0.521 |  |
|  | Precision | 0.465 | 0.408 |  |
|  | Recall | 0.800 | 0.721 |  |
| **RFS** | **Radiomic** |  |  |  |
|  | Youden Index | 0.509 |  |  |
|  | Accuracy | 0.773 | 0.705 |  |
|  | Accuracy 95% CI | 0.662-0.862 | 0.619-0.781 |  |
|  | No Information Rate | 0.520 | 0.561 |  |
|  | P-value (Acc > NIR) | 0.000 | 0.000 |  |
|  | Sensitivity | 0.667 | 0.690 |  |
|  | Specificity | 0.872 | 0.716 |  |
|  | PPV | 0.828 | 0.656 |  |
|  | NPV | 0.739 | 0.747 |  |
|  | F1 Score | 0.738 | 0.672 |  |
|  | Precision | 0.828 | 0.656 |  |
|  | Recall | 0.667 | 0.690 |  |
|  | **Combined** |  |  |  |
|  | Youden Index | 0.489 |  |  |
|  | Accuracy | 0.747 | 0.667 |  |
|  | Accuracy 95% CI | 0.633-0.841 | 0.579-0.746 |  |
|  | No Information Rate | 0.520 | 0.561 |  |
|  | P-value (Acc > NIR) | 0.000 | 0.008 |  |
|  | Sensitivity | 0.639 | 0.690 |  |
|  | Specificity | 0.846 | 0.649 |  |
|  | PPV | 0.793 | 0.606 |  |
|  | NPV | 0.717 | 0.727 |  |
|  | F1 Score | 0.708 | 0.645 |  |
|  | Precision | 0.793 | 0.606 |  |
|  | Recall | 0.639 | 0.690 |  |
| **Recurrence** | **Radiomic** |  |  |  |
|  | Youden Index | 0.358 |  |  |
|  | Accuracy | 0.653 | 0.462 |  |
|  | Accuracy 95% CI | 0.535-0.76 | 0.375-0.551 |  |
|  | No Information Rate | 0.640 | 0.697 |  |
|  | P-value (Acc > NIR) | 0.457 | 1.000 |  |
|  | Sensitivity | 0.926 | 0.925 |  |
|  | Specificity | 0.500 | 0.261 |  |
|  | PPV | 0.510 | 0.352 |  |
|  | NPV | 0.923 | 0.889 |  |
|  | F1 Score | 0.658 | 0.510 |  |
|  | Precision | 0.510 | 0.352 |  |
|  | Recall | 0.926 | 0.925 |  |
|  | **Combined** |  |  |  |
|  | Youden Index | 0.337 |  |  |
|  | Accuracy | 0.613 | 0.508 |  |
|  | Accuracy 95% CI | 0.494-0.724 | 0.419-0.596 |  |
|  | No Information Rate | 0.640 | 0.697 |  |
|  | P-value (Acc > NIR) | 0.729 | 1.000 |  |
|  | Sensitivity | 0.926 | 0.900 |  |
|  | Specificity | 0.438 | 0.337 |  |
|  | PPV | 0.481 | 0.371 |  |
|  | NPV | 0.913 | 0.886 |  |
|  | F1 Score | 0.633 | 0.526 |  |
|  | Precision | 0.481 | 0.371 |  |
|  | Recall | 0.926 | 0.900 |  |

**Supplementary Table 8:** Brier scores for the validation and external test set for each prediction model.

| Endpoint | Model | Validation Set | External Test Set |
| --- | --- | --- | --- |
| Recurrence | Radiomics only | 0.222 | 0.220 |
|  | Combined | 0.224 | 0.197 |
| RFS | Radiomics only | 0.292 | 0.289 |
|  | Combined | 0.295 | 0.291 |
| OS | Radiomics only | 0.310 | 0.312 |
|  | Combined | 0.311 | 0.315 |

**Supplementary Figure 1.** Calibration curves for validation and external test sets for each prediction model. The data is divided into bins, with the *y*-axis representing the distribution of positive cases in each bin while the *x*-axis the probability as predicted by the classifier. The closer the resulting calibration curve is to the reference line. the better the model's predictions reflect the actual class distribution in the dataset.


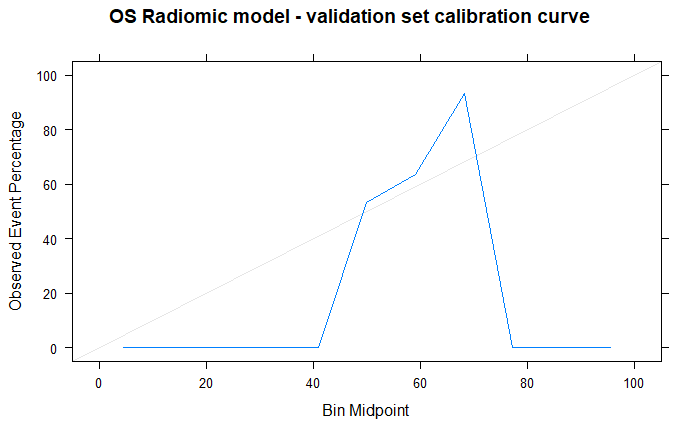

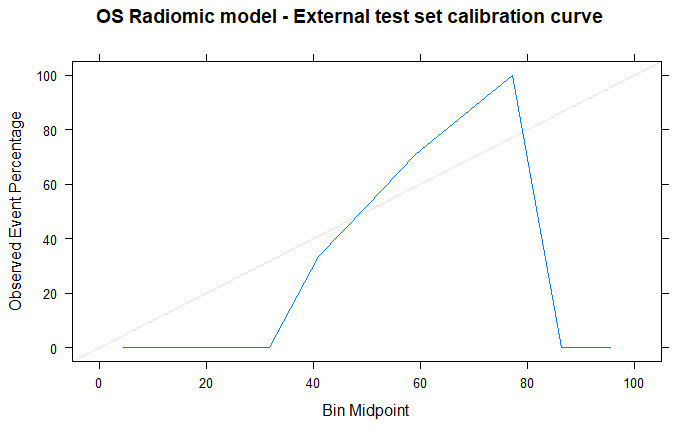


#


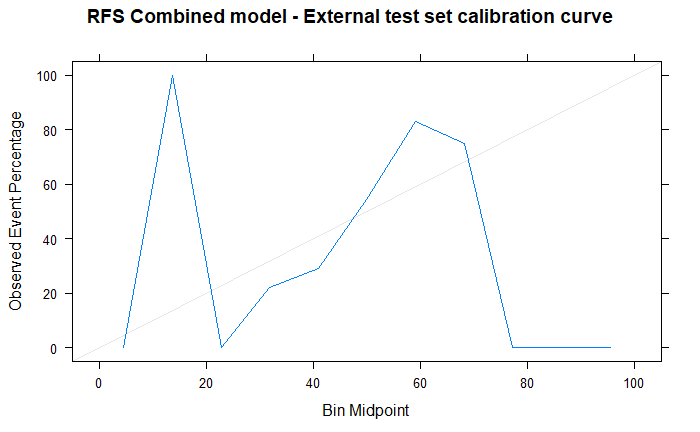

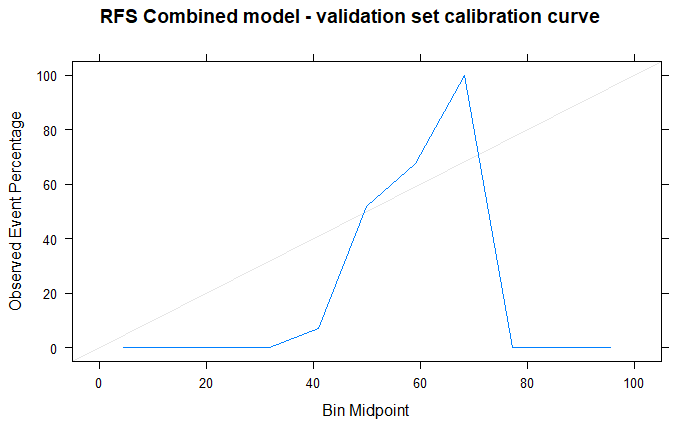

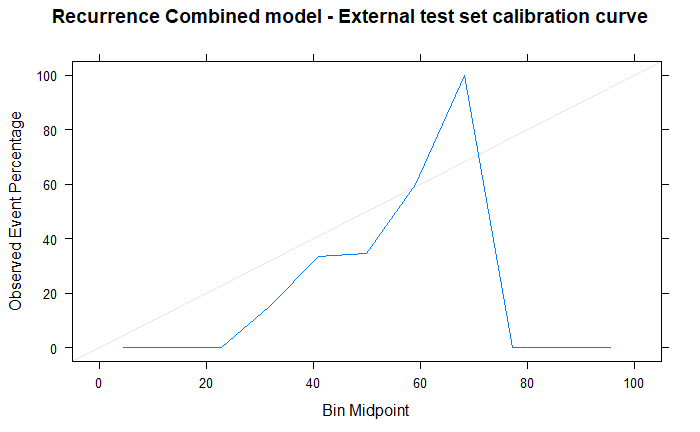

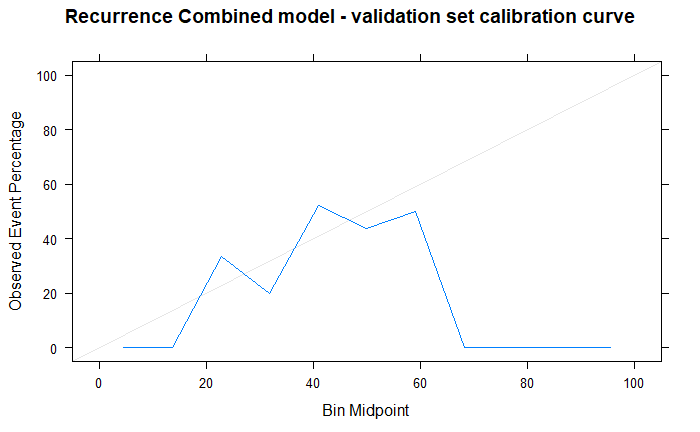

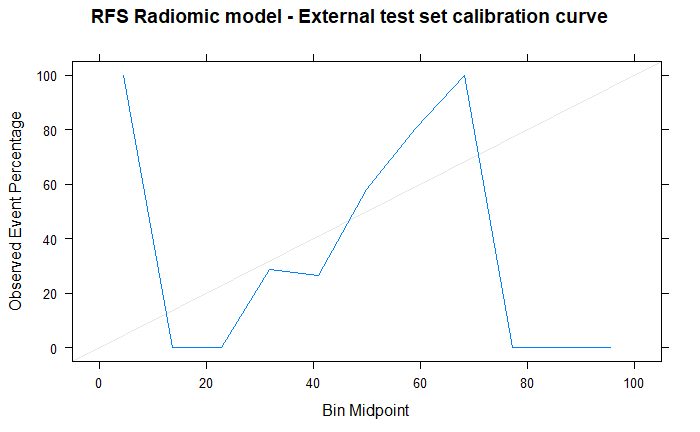

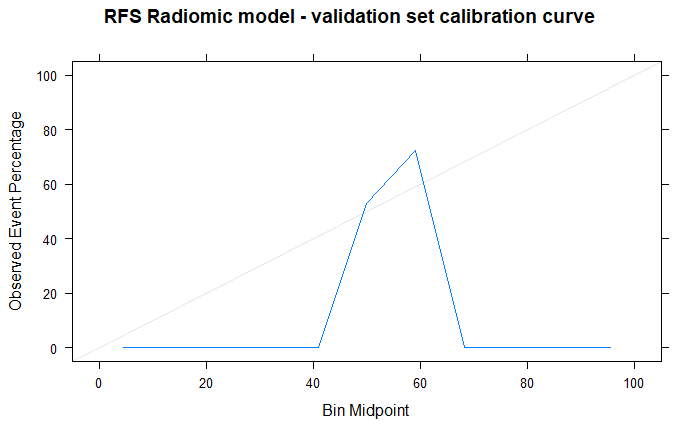

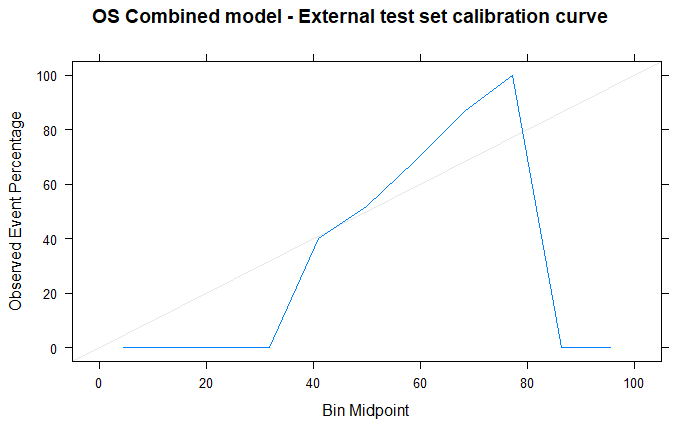

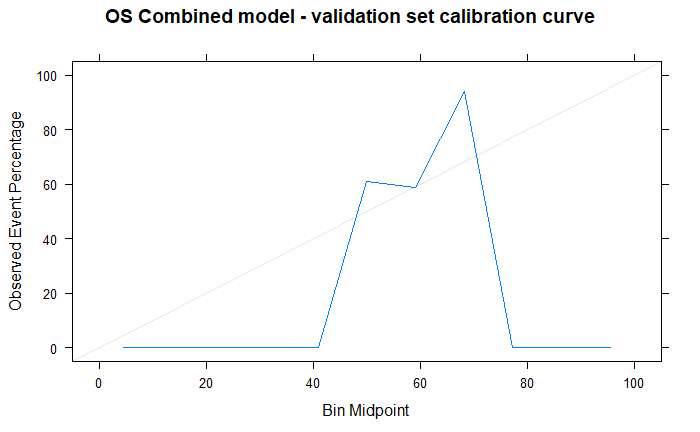

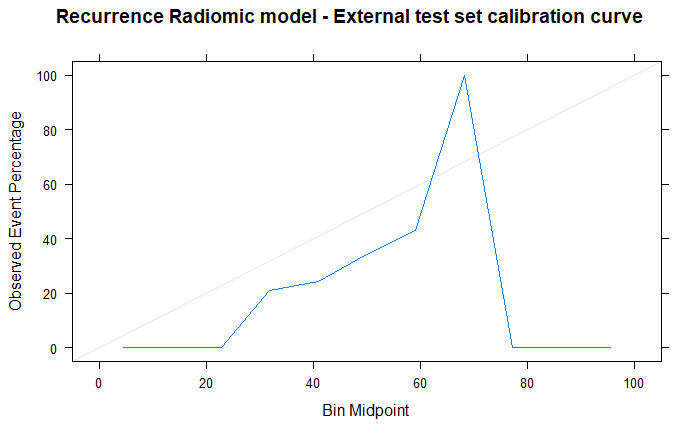

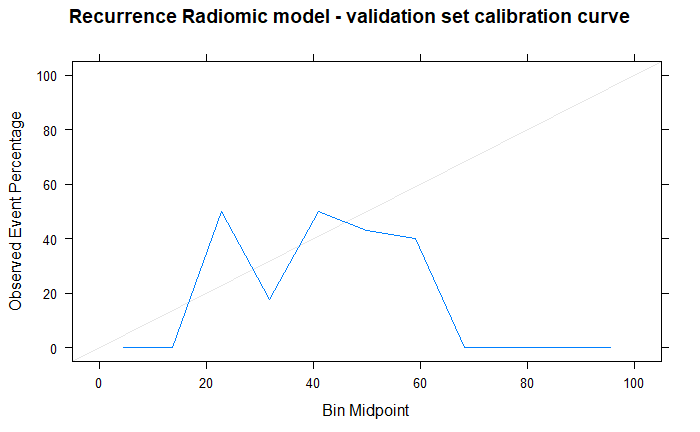


**Supplementary Material References:**

1 van Buuren S, Groothuis-Oudshoorn K. mice: Multivariate imputation by chained equations in R. *J Stat Softw* 2011; **45**: 1–67.

2 Choonghyun R. alookr: [1] Ripley, B., Venables, W. & Maintainer, ]. Package ‘nnet’ NeedsCompilation yes. (2021).Model Classifier for Binary Classification. 2021. https://github.com/choonghyunryu/alookr/issues (accessed Aug 12, 2021).

3 Friedman J, Hastie T, Tibshirani R. Regularization paths for generalized linear models via coordinate descent. *J Stat Softw* 2010; **33**: 1–22.

4 Venables WN, Ripley BD. Generalized Linear Models. In: Modern Applied Statistics with S. New York, NY: Springer New York, 2002: 183–210.

5 Guyon I, Weston J, Barnhill S, Vapnik V. Gene selection for cancer classification using support vector machines. *Mach Learn* 2002; **46**: 389–422.

6 Battiti R. Using Mutual Information for Selecting Features in Supervised Neural Net Learning. *IEEE Trans Neural Networks* 1994; **5**: 537–50.

7 Kursa MB, Rudnicki WR. Feature selection with the boruta package. *J Stat Softw* 2010; **36**: 1–13.

8 Karatzoglou A, Hornik K, Smola A, Zeileis A. kernlab - An S4 package for kernel methods in R. *J Stat Softw* 2004; **11**: 1–20.

9 Venables WN, Ripley BD. Classification. In: Modern Applied Statistics with S. New York, NY: Springer New York, 2002: 331–51.

10 Breiman L. Random forests. *Mach Learn* 2001; **45**: 5–32.

11 Chen T, Guestrin C. XGBoost: A scalable tree boosting system. In: Proceedings of the ACM SIGKDD International Conference on Knowledge Discovery and Data Mining. New York, NY, USA: ACM, 2016: 785–94.

12 Mevik B-H, Wehrens R. Introduction to the pls Package. Help Sect. ‘pls’ Packag. RStudio Softw. 2015; : 1–23.

13 Riplley B, Venables B, Bates DM, Firth D, Hornik K, Gebhardt A. Package ‘MASS’. Support Functions and Datasets for Venables and Ripley’s MASS. Doc. Free. available internet http//www. r-project. org. 2018; : 169.

14 Michal Majka. High performance implementation of the Naive Bayes algorithm. R package naivebayes. Version 0.9.7. 2019. https://majkamichal.github.io/naivebayes/ (accessed Aug 13, 2021).

15 He H, Bai Y, Garcia EA, Li S. ADASYN: Adaptive synthetic sampling approach for imbalanced learning. In: 2008 IEEE International Joint Conference on Neural Networks (IEEE World Congress on Computational Intelligence). 2008: 1322–8.

16 Figueroa RL, Zeng-Treitler Q, Kandula S, Ngo LH. Predicting sample size required for classification performance. *BMC Med Informatics Decis Mak 2012 121* 2012; **12**: 1–10.

17 Zhang Y, Oikonomou A, Wong A, Haider MA, Khalvati F. Radiomics-based Prognosis Analysis for Non-Small Cell Lung Cancer. *Sci Rep* 2017; **7**: 1–8.

18 Lambin P, Leijenaar RTH, Deist TM, *et al.* Radiomics: The bridge between medical imaging and personalized medicine. Nat. Rev. Clin. Oncol. 2017; **14**: 749–62.

19 Park JE, Kim D, Kim HS, *et al.* Quality of science and reporting of radiomics in oncologic studies: room for improvement according to radiomics quality score and TRIPOD statement. *Eur Radiol 2019 301* 2019; **30**: 523–36.

20 Sanduleanu S, Woodruff HC, Jong EEC de, *et al.* Tracking tumor biology with radiomics: A systematic review utilizing a radiomics quality score. *Radiother Oncol* 2018; **127**: 349–60.
